# Association between dry mouth and dry eyes: a systematic literature review of clinical evidence

**DOI:** 10.1007/s00296-025-05958-5

**Published:** 2025-08-28

**Authors:** Wiktoria Potocka, Zainab Assy, Mirjam M. A. van Tilborg, Floris J. Bikker, Marja L. Laine

**Affiliations:** 1 Department of Oral Biochemistry, Academic Centre for Dentistry Amsterdam, University of Amsterdam and VU University Amsterdam, Gustav Mahlerlaan, 3004, Amsterdam, 1081 LA The Netherlands; 2 Department of Periodontology, Academic Centre for Dentistry Amsterdam, University of Amsterdam and VU University Amsterdam, Gustav Mahlerlaan, 3004, Amsterdam, 1081 LA The Netherlands; 3 Research Group Technology for Healthcare Innovations, Research Centre Health and Sustainable Life, HU University of Applied Sciences, Utrecht, The Netherlands

**Keywords:** Xerostomia, Keratoconjunctivitis sicca, Dry eye syndromes, Sjögren's syndrome, Polypharmacy, Geriatrics

## Abstract

Sicca syndrome (dry mouth and dry eyes) occurs predominantly due to the side effects of medication, systemic diseases (Sjögren’s disease), and radiotherapy of the head and neck region. Sicca complaints decrease the quality of life, cause sleep disturbances, and affect overall health. This systematic literature review investigates the correlation and/or association between dry mouth and dry eyes. A comprehensive search was conducted through PubMed and Web of Science databases up to November 2024. English-language research studies investigating the association and/or correlation between dry mouth and dry eyes were included. Study quality was assessed using NIH quality assessment tools. Data on publication details, participant characteristics, assessment methods, and outcomes was extracted and synthesised based on the type of outcome (objective and/or subjective assessments) and cohort type. Out of 863 records, 27 studies met the eligibility criteria (published 1989–2024), including three observational cohort (*n* = 942), 15 cross-sectional (*n* = 6165), and nine case-control (*n* = 1326 patients, *n* = 841 controls). 13 studies focused on symptoms in Sjögren’s disease, seven in other patient groups, and eight in the general population. Significant correlation and/or association between oral and ocular objective assessments was found in 10 out of 27 studies, and between subjective assessments in 18 out of 19. Within that, 10 out 10 studies assessing self-reported subjective dry mouth and eyes found significant associations. This systematic review provides a general overview of the relationship between dry mouth and dry eyes. It also highlights the need for standardised diagnostic guidelines for dry mouth and dry eyes. PROSPERO registration: CRD42024555796.

**Supplementary Information:**

The online version contains supplementary material available at 10.1007/s00296-025-05958-5.

## Introduction

Sicca syndrome is a condition characterized by dryness of mouth and eyes. Dry mouth is estimated to affect between 5.5% and 46% of the population [1–4], and dry eyes affect from 3.5 to 61% of the population [5–8], depending on the population cohort and assessment methods used. The prevalence of dry mouth [9, 10] and dry eyes [5, 11, 12] increases with age and is higher in women compared to men. Risk factors for dry mouth and dry eyes include polypharmacy, side effects of medication (such as antidepressants, antihistamines, opiates, diuretics) [5, 13–15], as well as chemotherapy and radiation therapy [16, 17]. Furthermore, autoimmune diseases (e.g. Sjögren’s disease (SjD)), endocrine diseases (e.g. diabetes mellitus), neurological disorders (e.g. Parkinson’s disease), genetic and developmental diseases (e.g. cystic fibrosis), connective tissue diseases (e.g. rheumatoid arthritis (RA)) [18–21], chronic stress [12, 22, 23], and eating disorders (e.g. anorexia nervosa) [18, 24] can result in sicca syndrome. Moreover, meibomian gland dysfunctions and contact lens use can lead to dry eyes [25].

Dry mouth can be assessed in an objective (hyposalivation) or a subjective manner (xerostomia) [26], and can occur independently or simultaneously, the latter known as hyposalivation-induced xerostomia [27]. Hyposalivation can increase the risk of oral infections, tooth wear and caries, and negatively impact other aspects of oral health [14, 28]. Treatment is dependent on the functioning of the glands: if the glands are intact, options include stimulation of saliva secretion by lozenges, chewing gum, pilocarpine, or cevimeline [28, 29, 30]. In contrast, if the integrity of the glands is compromised, artificial saliva substitutes like gels, sprays, and mouthwashes are recommended [31].

Dry eyes are characterised by a loss of tear film homeostasis [32]. The condition, sometimes called dry eye disease (DED) can be categorised as aqueous-deficient, caused by insufficient tear production, or as hyper-evaporative, caused by increased evaporation due to deficient lipid layer. Both forms can occur simultaneously [11]. Treatment options for dry eyes includes eyelid hygiene, use of artificial tears (eyes drops) or ointments, anti-inflammatory drugs, topical corticosteroids, cyclosporin A, tacrolimus and punctal plugs [11, 33].

The constant discomfort caused by feeling of dry mouth [34, 35] and dry eyes [36–39] can have a detrimental effect on one’s wellbeing and health, and consequently impair the quality of life. However, there are no clear guidelines for diagnosis in the clinical setting, due to availability of various methodologies (objective and subjective) that can be used to assess dry mouth [40] and dry eyes [41]. Further, there is little understanding why do these symptoms co-occur, if the manifestation of one can causes the other symptoms to develop, and if there is an association between their prevalence.

Better understanding of the co-occurrence and subsequently correlation and/or association between dry mouth and dry eyes will help with more effective diagnosis, establishing of predictive factors and optimal treatments. This research aimed to review the current literature to identify if there is an association between dry mouth and dry eyes, and if so, the nature of the relationship. Additionally, the secondary aim was to better understand the clinical implications of this relationship.

## Methods

### Protocol registration

The research protocol was registered in the international Prospective Register of Systematic Reviews (PROSPERO; CRD42024555796) on the 17th of June 2024 [42]. An amendment to the protocol was made on the 8th of October 2024. Preferred Reporting Items for Systematic reviews and Meta-Analyses (PRISMA) 2020 Statement guidelines [43] and additional guidelines [44] were followed in the preparation of this manuscript.

### Eligibility criteria

Study eligibility inclusion criteria included: research studies published in English language that specifically investigate the correlation and/or association between dry mouth and dry eyes. Exclusion criteria included:

- studies not directly describing the correlation and/or association between dry mouth and dry eyes,
- studies that concentrated on only one of the conditions,
- in vitro studies,
- animal studies,
- experiments without direct relevance to the human conditions,
- studies with participants under the age of 18,
- randomized clinical trial studies,
- narrative, scoping, and systematic reviews, meta-analyses, interviews, case reports, meeting/conference abstracts and publications, preprints, opinion articles, letters to the editor and book chapters.

### Information sources, search strategy and selection process

A literature search using PubMed and Web of Science Core Collection was conducted on the 4th of June, and the final search on the 18th of November 2024 by two independent reviewers (WP, ZA). The search terms are presented in Table S1. Duplicates were removed using reference manager Zotero (Corporation for Digital Scholarship, Windows (v7.05)). All potentially eligible records were screened by two reviewers (WP, ZA) based on the title and abstract. For all eligible records, full text was obtained and screened by two reviewers (WP, ZA). If not available, the authors were contacted to obtain the full text (conditional on contact details availability). Any discrepancies were resolved through open discussion until an agreement was reached by two reviewers (WP, ZA). In case of any disagreements, third reviewer was consulted (MLL).

### Data collection process

After full report screening, eligible studies were selected for the data extraction process. For each included study, following information was recorded by one reviewer (WP):

- first author, publication year, country of study population, and study type,
- participant characteristics (total number, sex, and age),
- oral and ocular assessments performed (relating to dry mouth and dry eyes),
- outcome measures.

In case of discrepancies and queries, authors of studies were contacted for support, and the provided information was supplemented in the table. For any n- and %-values, if either was missing, the value was calculated based on the provided information.

### Effect measures

Descriptions of the results regarding dry mouth and dry eyes were reported in the outcome measures. To assess dry mouth, results from the assessments listed below were reported:

- salivary flow rate measurements,
- salivary gland assessments,
- validated dry mouth questionnaires,
- self-reported symptoms/questionnaires.

To assess dry eyes, results from the assessments listed below were reported:

- tear assessments,
- meibomian gland assessments,
- ocular staining,
- validated dry eyes questionnaires,
- self-reported symptoms/questionnaires.

Furthermore, for any correlations and/or associations between the assessments that were recorded, type of statistical test, results and significance were recorded. In case of missing data, it was annotated as “not reported” (NR). For any n- and %-values, if either was missing, the value was calculated based on the provided information.

### Risk of bias assessment

Two reviewers (WP, ZA) independently assessed the risk of bias of the included studies using the NIH study quality assessment tools [45], including “Quality Assessment Tool for Observational Cohort and Cross-Sectional Studies” and “Quality Assessment of Case-Control Studies”. These tools have been developed to evaluate the internal validity of a study and test for potential flaws in study methods or implementation. Differences in rating between reviewers were resolved by discussion between three reviewers (WP, ZA, MLL). If the question was not applicable to the type of research, “not applicable” (NA) was used as an answer. If researchers did not report on the item, “NR” was used as an answer. The total score was counted by dividing the number of “yes” responses by the number of total questions (the totals were adjusted for “NA” responses – number of NA responses was removed from the total; “NR” were counted as a “no”). This value was then multiplied by 100% and rounded to the first decimal place. The studies were rated as “poor” when ≤ 33.3%, as “fair” between > 33.3% and ≤ 66.7% and as “good” >66.7%.

### Synthesis methods

The studies which were selected for the data extraction and undergone risk of bias assessment were used for the synthesis. Information such as type of statistical test used, results and significance were reported. Due to the extensive heterogeneity in outcome measures and reporting, it was not possible to perform a meta-analysis or statistical synthesis.

Given the heterogeneity of the studies being investigated, they were categorized based on the type of assessment (objective versus objective, subjective versus subjective, objective versus subjective). Additionally, results were synthesised based on the cohort (general population, elderly population, SjD, other systemic diseases, cancer and transplant patients).

## Results

### Study selection

**Fig. 1:**
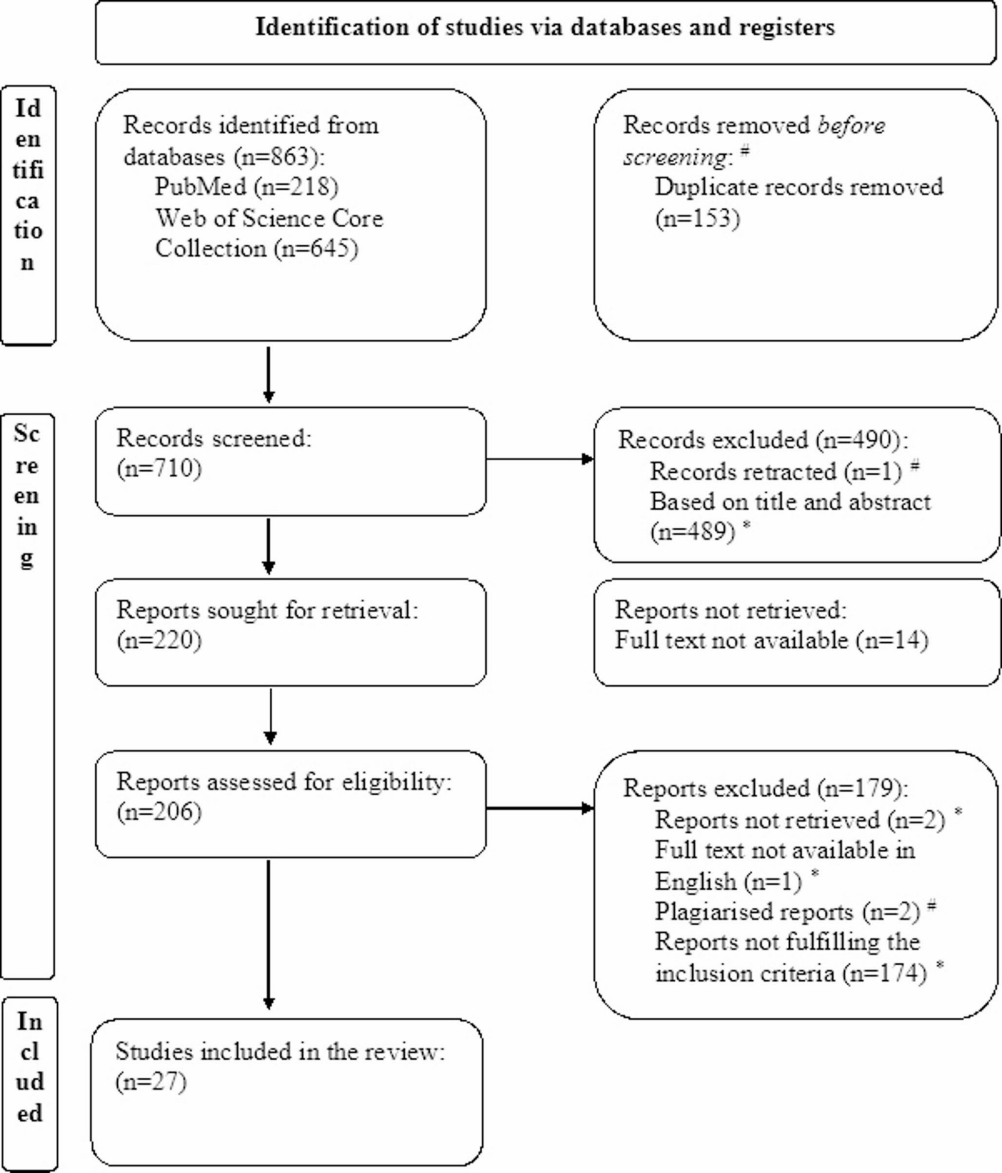
Flow chart for the systematic review process. ^#^ Records and reports excluded using Zotero. ^*^ Records and reports excluded manually

A total of 863 records fulfilled the search criteria and undergone screening, including records from PubMed (*n* = 218) and Web of Science Core Collection (*n* = 645) (Fig. 1). After removing 153 duplicates, 710 records remained. Based on title and abstract, 490 records were excluded and 220 were sought for retrieval. 14 reports were not retrieved due to the digital text being unavailable (no contact details available/ no response from authors received), and accordingly, 206 reports were assessed for eligibility. After the full report screening, 27 studies met the eligibility criteria. Five studies, while appeared to meet the eligibility criteria, were excluded. In the publication by Hora et al. [46], Fisher’s exact test was used to asses correlation and association. Similarly, Daniels and Witcher’s [47] study was excluded, as it applied χ^2^ for estimating association. On the other hand, Dejaco et al. [48] studied the application of B-mode ultrasonography and real-time sonoelastography of salivary glands with dry eye measurements, however these parameters did not assess the association. Two studies were excluded [49, 50], as they reused patient data from previously published studies [51, 52], that were included in the synthesis.

**Table 1:** Methodology used in the included studies to assess dry mouth

	References
**Objective assessments**	
***Salivary flow rate assessments***	
Unstimulated whole saliva (UWS)	[51–68]
Stimulated whole saliva (SWS)	[52, 54, 55, 57, 61, 62, 64, 66, 68, 69]
Labial salivary gland (LSG) flow rate, minor salivary gland (MSG) flow rate	[64, 70]
Parotid flow rate, submandibular/sublingual glands flow rate	[52, 67, 71]
***Salivary gland assessments***	
LSG biopsy	[58, 63, 72–74]
MSG biopsy	[59, 65, 66]
MSG score (count)	[70]
Salivary gland scintigraphy (SGS)	[65, 66, 71, 72]
**Subjective assessments**	
***Validated questionnaires***	
Clinical Oral Dryness Score (CODS) ^#^	[61]
Xerostomia Inventory (XI) ^*^, Summated Xerostomia Inventory (S-XI) ^†^	[61, 62, 75]
Sicca Symptoms Inventory (SSI) oral domain score ^‡^	[68]
***Subjective complaints***	
Presence/diagnosis of xerostomia symptoms, xerostomia frequency	[58, 61]
Self-reported symptoms/questionnaires	[52–57, 60–64, 66, 67, 69–71, 74, 76, 77]
**Subjective and objective assessments**	
***Joint score***	
Oral sensitivity ^§^	[56]

^#^ Osailan et al. [78], ^*^ Thomson et al. [79], ^†^ Thomson et al. [80], ^‡^ Raymond et al. [81]. , ^§^ Bezzina et al. [56]

**Table 2:** Methodology used in the included studies to assess dry eyes

	References
**Objective assessments**	
***Tear assessments***	
Schirmer I test (without anaesthesia), Schirmer II test (with anaesthesia)	[50, 52, 53, 56, 58–62, 65–67, 69–74]
Tear break-up time (TBUT)	[51, 52, 59, 61, 62, 66, 74]
***MG assessments***	
Meibomian gland (MG) dysfunction	[74]
***Ocular staining***	
Ocular staining score (OSS), Rose Bengal staining, Lissamine Green staining, Fluorescein staining, Marx Line staining, van Bijsterveld score	[51, 52, 59, 61, 62, 66, 67, 70, 71, 73, 74]
***Presence/diagnosis of***:	
Keratoconjunctivitis sicca (KCS) symptoms	[59, 63, 69, 71, 74]
**Subjective assessments**	
***Validated questionnaires***	
Ocular Surface Disease Index (OSDI) ^#^	[54, 61, 62, 66, 70]
McMonnies Dry Eye Questionnaire (MDEQ) ^*^	[61]
Sicca Symptoms Inventory (SSI) ocular domain score ^‡^	[68]
***Subjective complaints***	
Self-reported symptoms/questionnaires	[52–60, 62–64, 66, 67, 69, 71, 73–77]
**Subjective and objective assessments**	
***Joint score***	
Ocular sensitivity ^§^	[56]

^#^ Schiffman et al. [82], ^*^ McMonnies and Ho [83], ^‡^ Raymond et al. [81], ^§^ Bezzina et al. [56]

### Description of methodology assessing oral and ocular dryness

The following methods were applied to assess oral (Table 1) and ocular dryness (Table 2). The objective dry mouth assessments that were carried out (*n* = 47): unstimulated whole saliva (UWS) (*n* = 18), stimulated whole saliva (SWS) (*n* = 10), labial salivary gland (LSG) flow rate and minor salivary gland (MSG) flow rate (*n* = 2), parotid flow rate and submandibular/sublingual glands flow rate (*n* = 3), LSG biopsy (*n* = 5), MSG biopsy (*n* = 3), MSG score (count) (*n* = 1), salivary gland scintigraphy (SGS) (*n* = 4). The subjective dry mouth assessments that were carried out (*n* = 25): Xerostomia Inventory (XI) and summated XI (S-XI) (*n* = 3), Sicca Symptoms Inventory (SSI) oral domain score (*n* = 1), presence/diagnosis of xerostomia symptoms and xerostomia frequency (XF) (*n* = 2), self-reported symptoms/questionnaires (*n* = 19). Joint assessment for both objective and subjective assessments was carried out in a form of oral sensitivity (*n* = 1).

The objective dry eye assessments that were carried out in (*n* = 37): Schirmer I test (without anaesthesia) and Schirmer II test (with anaesthesia) (*n* = 14), tear break up time (TBUT) (*n* = 7), meibomian gland (MG) dysfunction (*n* = 1), ocular staining (*n* = 11), presence/diagnosis of keratoconjunctivitis sicca (KCS) symptoms (*n* = 5). The subjective dry eye assessments that were carried out in (*n* = 28): Ocular Surface Disease Index (OSDI) (*n* = 5), McMonnies Dry Eye Questionnaire (MDEQ) (*n* = 1), SSI ocular domain score (*n* = 1), self-reported symptoms/questionnaires (*n* = 21). Joint assessment for both objective and subjective assessments was carried out in a form of ocular sensitivity (*n* = 1).

**Table 3:** Main characteristics of studies investigating the objective and subjective correlation and/or association between dry mouth and dry eyes

First author, publication year	Country of study population	Study type	Population	Participant characteristics [total (*n*), female/male (*n*), age (y)]	Outcomes [comparisons, results, *p*-value]	Statistical analysis
Bassim et al. [53], 2015	USA	Cross-sectional	cGVHD patients	*n* = 212, F 99/M 113 • (median ± range) 48 ± 18–70 y	• Correlation between self-reported dry mouth and self-reported dry eyes (*r* = 0.32, *p* < 0.01).	Spearman’s rank correlation coefficient
Baudin et al. [54], 2023	France	Observational cohort	DTC patients receiving ^131^I-therapy with ^131^I-activity of 1.1 GBq or 3.7 GBq	*n* = 136, F 97/M 39 47.1 ± 14.1 y • *n* = 44, F/M NR NR y • *n* = 92, F/M NR NR y	• No correlation between UWS ≤ 0.25 mL/min, SWS ≤ 1.00 mL/min, UWS, or SWS and OSDI. • Correlation between self-reported dry mouth and self-reported dry eyes (NR, *p* = 0.01). • No correlation between UWS, UWS ≤ 0.25 mL/min, SWS ≤ 1.00 mL/min, or SWS and self-reported dry eyes. Correlation between self-reported dry mouth and OSDI (NR, *p* = 0.01).	Pearson correlation coefficient, ANOVA, χ² test
Bergdahl [55], 2000	Sweden	Cross-sectional	Dental clinic attendees	*n* = 1427, F 758/M 669 • 10 groups in the range 20–69 y	• Individuals with self-reported dry mouth had higher OR of self-reported dry eyes (*n* = 59, OR: 1.65, 95% CI: 1.04–2.62, *p* < 0.05).	Multiple logistic regression
Bezzina et al. [56], 2017	UK	Cross-sectional	pSjD patients	*n* = 688, F 651/M 37 • 58 ± 12.5 y	• Correlation between oral and ocular sensitivity (*r* = 0.35, *p* < 0.001).	Spearman’s bivariate correlation
Billings et al. [57], 1996	USA	Cross-sectional	Community-dwelling adults	*n* = 710, F 484/M 226 • range 19–88 y	• Association between xerostomia and self-reported dry eyes in F (crude OR: 3.2, 95% CI: 1.8–5.5; age-adjusted OR: 2.7, 95% CI: 1.5–4.8, *p* < 0.05) and M (crude OR: 4.5, 95% CI: 1.9–11.0; age-adjusted OR: 6.7, 95% CI: 2.3–19.0, *p* < 0.05).	Logistic regression analysis
Błochowiak [58], 2024	Poland	Cross-sectional	SjD patients	*n* = 50, F 43/M 7 • 52 ± 10.9 y	• Correlation between UWS and self-reported dry eyes (r = NR, *p* = 0.02).	Spearman’s rank correlation coefficient
Chiu et al. [72], 2020	Taiwan	Case-control	SjD patients and non-SjD sicca patients	• *n* = 247, F 219/M 28 56.6 ± 14.7 y • *n* = 268, F 221/M 47 54.7 ± 15 y	• No association between SGS and Schirmer I test.	χ2 test with Cramer’s V
Da Cunha et al. [59], 2022	France	Cross-sectional	pSjD patients and non-SjD sicca patients; later divided into “severe KCS” and “non-severe KCS”	• *n* = 253, F 245/M 8 56.6 ± 13 y • *n* = 108, F 98/M 10 56.4 ± 13.8 y; divided into: • *n* = 94, F 90/M 4 58 ± 13.5 y • *n* = 159, F 155/M 4 55.9 ± 14.1 y	pSjD patients • No association between abnormal UWS and conjunctival OSS, corneal OSS, Schirmer I test ≤ 5 mm/5 min or TBUT ≤ 10 s. Severe KCS patients • No association between abnormal UWS and KCS.	Multivariate analysis
Fernandez Castro et al. [73], 2018	Spain	Cross-sectional	pSjD patients with not severe dry eyes symptoms and severe/very severe dry eyes symptoms	• *n* = 59, F 57/M 2 49.3 ± 13.9 y • *n* = 378, F 359/M 19 50.6 ± 12.7 y	• No association between LSG biopsy focus score ≥ 1 and self-reported dry eyes. No association between unknown LSG biopsy focus score and self-reported dry eyes.	Multivariate analysis
Gilboe et al. [60], 2001	Norway	Case-control	Patients with SLE or RA with sicca symptoms and a suspicion of aSjD, and healthy controls	• *n* = 81, F 72/M 9 (mean ± range) 44 ± 20–70 y • *n* = 81, F 72/M/9 44 ± 22–69 y • *n* = 81, F 72/M 9 44 ± 20–70 y	SLE patients • No correlation between UWS and Schirmer I test of left and right eyes.	Pearson correlation coefficient
Hynne et al. [61], 2022	Norway	Cross-sectional	65-year-old population	*n* = 150, F 82/M 65 65 ± 0 y	• No correlation between UWS ≤ 0.1 mL/min and TBUT ≤ 10 s, Schirmer I test ≤ 10 mm/5 min and OSS ≥ 1. No correlation between SWS ≤ 0.7 mL/min and TBUT ≤ 10 s, Schirmer I test ≤ 10 mm/5 min and OSS ≥ 1. • Correlation between S-XI and MDEQ (*r* = 0.36, *p* < 0.001), and OSDI (*r* = 0.36, *p* < 0.001). S-XI > 10 and OSDI > 12 (*r* = 0.22, *p* = 0.01), and MDEQ > 10.5 (*r* = 0.22, *p* = 0.01). Correlation between XF and MDEQ (*r* = 0.42, *p* < 0.001), and OSDI (*r* = 0.28, *p* < 0.001). Correlation between XF > 3 and MDEQ > 10.5 (*r* = 0.21, *p* = 0.01), but not OSDI > 12. • No correlation between S-XI > 10 and TBUT ≤ 10s, Schirmer I test ≤ 10 mm/5 min or OSS ≥ 1. Correlation between XF > 3 and Schirmer I test ≤ 10 mm/5 min (*r* = 0.18, *p* = 0.03), and OSS ≥ 1 (*r* = 0.18, *p* = 0.03), not TBUT ≤ 10 s. No correlation between UWS ≤ 0.1 mL/min and OSDI > 12, MDEQ > 10.5. No correlation between SWS ≤ 0.7 mL/min and OSDI > 12, MDEQ > 10.5.	Spearman’s rho correlation analysis with Bonferroni correction
Kalk et al. [52], 2002	the Netherlands	Cross-sectional	pSjD, aSjD and non-SjD patients	• *n* = 32, F 30/M 2 53 ± 14 y • *n* = 25, F 19/M 6 58 ± 14 y • *n* = 23, F 21/M 2 48 ± 12 y	SjD patients • Correlation between stimulated flow of submandibular/sublingual salivary glands and Schirmer I test (*r* = 0.29, *p* < 0.01).	Spearman’s rank correlation coefficient
Kasetsuwan et al. [76], 2012	Thailand	Cross-sectional	Elderly population	*n* = 625, F 392/M 233 65.3 ± 7.5 y	• Self-reported dry mouth is a risk ratio for self-reported dry eyes (adj. OR: 2.008, 95% CI: 1.2–3.3, *p* = 0.005).	Binary logistic regression
Lackner et al. [62], 2021	Austria	Observational cohort	pSjD patients	*n* = 123, F 113/M 10 60.1 ± 12.3 y	• Correlation between UWS and OSDI (*r* = − 0.431, *p* < 0.05), SWS and OSDI (*r* = − 0.532, *p* < 0.01). • No correlation between UWS and VAS-sicca-eyes, SWS and VAS-sicca-eyes. No correlation between XI and Schirmer I test, VAS-sicca-mouth and Schirmer I test.	Spearman’s rank correlation coefficient
Leite et al. [74], 2006	Brazil	Case-control	Patients undergone allogeneic HPCT and healthy controls	• *n* = 124, F 44/M 80 31.5 ± 12.1 y • *n* = 10, F/M NR NR y	• Self-reported dry mouth (OR: 3.93, 95% CI: 1.66, 9.34, *p* = 0.002) is a risk and predictive factor for self-reported dry eyes. • Altered LSG biopsy (OR: 4.14, 95% CI: 1.74, 9.84, *p* = 0.0013) is a risk and predictive factor for self-reported dry eyes.	Logistic regression analysis
Oxholm et al. [51], 1989	Denmark	Observational cohort	pSjD patients	*n* = 28, F 24/M 4 (mean ± range) 51 ± 32–71 y	• Correlation between UWS and Schirmer I test (*r* = − 0.54, *p* < 0.01), UWS and van Bijsterveld score (*r* = 0.52, *p* < 0.01).	Pearson correlation coefficient
Pedersen et al. [63], 1999	Denmark	Case-control	pSjD patients, healthy age-matched controls, and reference group	• *n* = 16, F 14/M 2 range 40–82 y • *n* = 14, F 13/M 1 range 39–70 y • *n* = 13, F 12/M 1 range 20–33 y	• Correlation between UWS and number of self-reported dry eye complaints (r = NR, *p* = 0.007).	Spearman’s rank correlation coefficient
Rasker et al. [71], 1990	UK	Case-control	SCL patients with suspicion of SjD and three healthy control groups	• *n* = 26, F 22/M 4 53.5 ± 10.2 y • *n* = 24, F/M NR 52.8 ± 10.3 y • *n* = 24, F/M NR NR y • *n* = 21, F/M NR 50 ± 12.2 y	• Correlation between self-reported dry mouth and self-reported dry eyes (r = NR, *p* < 0.00 L). • Correlation between self-reported dry mouth and Schirmer I test (r = NR, *p* < 0.05).	NR
Singh and Basu [70], 2022	India	Case-control	SJS, SjD patients and healthy controls	• *n* = 15, F/M NR 35.7 ± 10.9 y • *n* = 15, F/M NR 57.7 ± 19 y • *n* = 40, F/M NR 10–60 y split into groups: 20–30, 30–40, 40–50, 50–60 y	SJS patients • No correlation between MSG flow rate and Schirmer I test. SjD patients • No correlation between MSG flow rate and Schirmer I test.	Pearson correlation coefficient
Smidt et al. [64], 2011	Denmark	Cross-sectional	Elderly population	*n* = 668, F 389/M 279 75.5 ± 6.4 y	• Correlation between self-reported dry mouth and self-reported dry eyes (OR: 4.7; 95% CI: 2.7–8.3, *p* < 0.001). • No association between UWS, SWS or unstimulated LSG flow rate and self-reported dry eyes.	Linear regression analysis
Troncoso et al. [65]	Spain	Cross-sectional	Sicca patients with SjD or non-SjD	*n* = 142, F 127/M 15 56.1 ± 1.5 y • *n* = 84, F 78/M 6 NR y • *n* = 58, F 49/M 9 NR y	• No correlation between SGS parameters: qualitative evaluation, uptake ratio, time-activity curve or ejection fraction global and Schirmer I test.	Pearson correlation coefficient
Ture et al. [66], 2023	South Korea	Cross-sectional	pSjD patients	*n* = 66, F 66/M 0 49.2 ± 11.3 y	• No correlation between UWS and OSS. Correlation between SWS (*r* = − 0.453, *p* = 0.001), submandibular gland uptake ratio (*r* = − 0.378, *p* = 0.007), parotid gland uptake ratio (*r* = − 0.476, *p* < 0.000), submandibular gland ejection fraction (*r* = − 0.287, *p* = 0.043), parotid gland ejection fraction (*r* = − 0.362, *p* = 0.01), MSG biopsy focus score (*r* = 0.362, *p* = 0.013) and OSS. No correlation between UWS, SWS, submandibular gland uptake ratio, parotid gland uptake ratio, submandibular gland ejection fraction, parotid gland ejection fraction, or MSG biopsy focus score and TBUT. • Correlation between oral-VAS and ocular-VAS (*r* = 0.297, *p* = 0.015), but no correlation between oral-VAS and OSDI. • No correlation between oral-VAS and OSS and oral-VAS and. No correlation between UWS, SWS, submandibular gland uptake ratio, parotid gland uptake ratio, submandibular gland ejection fraction, parotid gland ejection fraction, or MSG biopsy focus score and ocular-VAS.	Spearman’s rank correlation coefficient
Villa and Abati [77], 2011	Italy	Cross-sectional	Dental clinic attendees	*n* = 601, F 204/M 296 (median ± IQR) 47 ± 32–63 y	• Self-reported dry eyes are a risk factor for self-reported dry mouth (OR: 2.6, 95% CI: 1.5–4.7, *p* < 0.05).	Logistic regression model
Wang et al. [75], 2020	Australia	Cross-sectional	Elderly population	*n* = 627, F 291/M 336 75 ± 7 y	• Correlation between S-XI and self-reported dry eye symptoms frequency (*r* = 0.379, *p* < 0.001), presence of symptomatic xerostomia and self-reported dry eye symptoms frequency (*r* = 0.187, *p* < 0.001).	Spearman’s rank correlation coefficient
Wangkaew et al. [69], 2006	Thailand	Case-control	Patients with RA, SLE, SCL and matched healthy controls for RA, SLE, SCL	• *n* = 50, F 47/M 3 46.6 ± 8.8 y • *n* = 50, F 50/M 0 39.1 ± 9.6 y • *n* = 50, F 37/M 13 46.6 ± 7.9 y • *n* = 50; F 32/M 18 45.5 ± 8.3 y • *n* = 50, F 36/M 14 39.1 ± 9.6 y • *n* = 50, F 33/M 17 46.2 ± 7.3 y	RA patients • Correlation between SWS and Schirmer I test (*r* = 0.39, *p* < 0.01). No correlation between SWS and KCS. • Correlation between self-reported dry mouth (*r* = 0.32, *p* < 0.05), or xerostomia (*r* = 0.32, *p* < 0.05) and self-reported dry eyes. • No correlation between self-reported dry mouth or xerostomia and Schirmer I test. Correlation between self-reported dry mouth (*r* = 0.54, *p* < 0.01), or xerostomia (*r* = 0.54, *p* < 0.01) and KCS. No correlation between SWS and self-reported dry eyes. SLE patients • No correlation between SWS and Schirmer I test. No correlation between SWS and KCS. • Correlation between self-reported dry mouth (*r* = 0.51, *p* < 0.01), or xerostomia (*r* = 0.39, *p* < 0.01) and self-reported dry eyes. • No correlation between SWS and self-reported dry eyes. Correlation between self-reported dry mouth (*r* = 0.30, *p* < 0.05), or xerostomia (*r* = 0.37, *p* < 0.01) and Schirmer I test. Correlation between self-reported dry mouth (*r* = 0.38, *p* < 0.01), xerostomia (*r* = 0.44, *p* < 0.01) and KCS. SCL patients • No correlation between SWS and Schirmer I test. Correlation between SWS and KCS (*r* = 0.38, *p* < 0.01). • No correlation between self-reported dry mouth, or xerostomia and self-reported dry eyes. • No correlation between SWS and self-reported dry eyes. No correlation between self-reported dry mouth, or xerostomia and Schirmer I test. Self-reported dry mouth or xerostomia and KCS.	Pearson correlation coefficient, Spearman’s rank correlation coefficient, Eta test
Wróbel-Dudzińska et al. [67], 2021	Poland	Case-control	Population attending dental and optometrist clinics, split into six groups based on their general health: A (controls), B-F (patients)	*n* = 642, F 405/M 237 50.2 ± 11.4 y • A: *n* = 156, F 92/M 64 45.1 ± 12.2 y • B: *n* = 109, F 67/M 42 49 ± 11.1 y • C: *n* = 103, F 86/M 17 50 ± 10.7 y • D: *n* = 115, F 78/M 37 52.3 ± 8.4 y • E: *n* = 84, F 45/M 39 51.7 ± 9.2 y • F: *n* = 75, F 37/M 38 57.7 ± 12.1 y	• Correlation between UWS and Schirmer I test (A: *r* = 0.53, B: *r* = 0.46, C: *r* = 0.67, D: *r* = 0.67, E: 0.68, F: *r* = 0.76, *p* < 0.001), parotid flow and Schirmer I test (A: *r* = 0.39, B: *r* = 0.41, C: *r* = 0.63, D: *r* = 0.65, E: *r* = 0.76, F: *r* = 0.76, *p* < 0.001).	Pearson correlation coefficient
Xin et al. [68], 2020	Hong Kong	Case-control	pSjD, aSjD patients and healthy controls	• *n* = 38, F 36/M 2 50.8 ± 8.5 y • *n* = 47, F 47/M 0 50 ± 10.5 y • *n* = 40, F 40/M 0 51.4 ± 7.4 y	• Correlation between UWS and SSI ocular domain score (*r* = − 0.26, *p* < 0.005), SWS and SSI ocular domain score (*r* = − 0.28, *p* < 0.005).	Spearman’s rank correlation coefficient

Values are reported as mean ± SD unless specified. More detailed information regarding the reported studies can be found in the Table S1

Abbreviations: ^131^*I-activity* activity after vectorized internal radioactive iodine therapy, *cGVHD* chronic graft versus host disease, *DTC* differentiated thyroid carcinoma, *F* female, *HPCT* haematopoietic progenitor cell transplantation, *KCS* keratoconjunctivitis sicca, *LSG* labial salivary gland (reported as LMSG), *M* male, *MDEQ* McMonnies dry eyes questionnaire, *MG* meibomian gland, *MSG* minor salivary gland, *NR* not reported, *OSDI* ocular surface disease index (0-100), *OR* odds ratio, *OSS* Ocular Staining Score (0–12), *pSjD* primary Sjögren’s disease (reported as pSS), *RA* rheumatoid arthritis, *SCL* systemic sclerosis, *SGS* salivary gland scintigraphy, *SJS* Stevens-Johnson syndrome, *SLE* systemic lupus erythematosus, *SjD* Sjögren’s disease (reported as SS), *aSjD* associated Sjögren’s disease (reported as sSS), *SSI* Sicca Symptoms Inventory, *SWS* stimulated whole saliva (reported as SWFS, SWSFR, SFR, Saxon(’s) test), *S-XI* summated xerostomia nventory (reported as Summated Xerostomia Inventory-Dutch Version), *TBUT* tear break up time (reported as FBUT, TFBUT, BUT), *UWS* unstimulated whole saliva (reported as UWSF, UWSFR, UFR), *XI* xerostomia inventory, *y* years

### Description of studies and participant characteristics

The studies that met the eligibility criteria were published between 1989 and 2024. Within the selected studies, three studies were identified as observational cohort studies, 15 as cross-sectional, and 9 as case-control (Table 3, Table S2). In total, there were 9274 participants, including case-control studies (*n* = 1326 patients and *n* = 841 controls), observational cohort (*n* = 942), and cross-sectional (*n* = 6165). The participants’ mean reported age ranged between 31.5 and 75.5 years old. Study populations included participant cohorts from 18 countries: Australia (*n* = 1), United States of America (*n* = 2), Brazil (*n* = 1), Hong Kong (*n* = 1), India (*n* = 1), South Korea (*n* = 1), Taiwan (*n* = 1), Thailand (*n* = 2), Austria (*n* = 1), Denmark (*n* = 3), France (*n* = 2), Italy (*n* = 1), the Netherlands (*n* = 1), Norway (*n* = 2), Poland (*n* = 2), Spain (*n* = 2), Sweden (*n* = 1) and United Kingdom (*n* = 2). All studies were single centre studies.

Four studies measured the outcomes in general population [55, 57, 67, 77], and four in the elderly population [61, 64, 75, 76]. Furthermore, 13 studies researched dry mouth and dry eyes in SjD, including 10 studies investigating primary SjD (pSjD) patients [51, 52, 56, 58, 59, 62, 63, 66, 68, 73], and two both pSjD and associated SjD (aSjD) patients [52, 68]. Three studies did not distinguish between pSjD and aSjD patients, and grouped them as one cohort [65, 70, 72]. Seven studies focused on other patient groups, including four describing systemic diseases [60, 69–71] and three transplantation or cancer patients [53, 54, 74].

### Risk of bias assessment

During the study quality assessment, out of 18 cohort and cross-sectional studies, five studies were rated as “poor” [57, 58, 64, 66, 76], 12 as “fair” [51, 52, 53, 55, 56, 59, 61, 62, 65, 73, 75, 77] and one as “good” [54] (Table S3). Out of nine case-control studies, six studies were rated as “poor” [63, 67, 69, 70, 71, 74], and three as “fair” [60, 68, 72] (Table S4). Studies with a “poor” quality assessment were not excluded, however the findings should be reviewed and analysed with caution.

**Table 4:** Matrix of objective oral and ocular assessments in the included studies, among which correlation and/or association analysis was performed. Bolded reference indicates at least one significant correlation between the assessments (sometimes multiple correlations were carried out, for example due to different patient groups). For non-significant results, only the available numerical values were included in this table

Ocular assessments Oral assessments	Schirmer I test		TBUT		OSS		van Bijsterveld score		Presence/diagnosis of KCS symptoms	
	*N* studies with significant results/total *N*	Ref.	*N* studies with significant results/total *N*	Ref.	*N* studies with significant results/total *N*	Ref.	*N* studies with significant results/total *N*	Ref.	*N* studies with significant results/total *N*	Ref.
UWS	2/5	[51, 59,– 61, 67]	0/3	[59, 61, 66]	0/3	[59, 61, 66]	1/1	[51]	0/1	[59]
SWS	1/2	[61, 69]	0/2	[61, 66]	1/2	[61, 66]	NR		1/1	[69]
Parotid gland flow rate	1/1	[67]	NR		NR		NR		NR	
Submandibular/sublingual glands flow rate	1/1	[52]	NR		NR		NR		NR	
MSG flow rate	0/1	[70]	NR		NR		NR		NR	
MSG biopsy	NR		0/1	[66]	1/1	[66]	NR		NR	
SGS	0/2	[65, 72]	0/1	[66]	1/1	[66]	NR		NR	

Abbreviations: *KCS* keratoconjunctivitis sicca, *LSG* labial salivary gland, *NR* no statistics for associations and/or correlations reported, *OSS* ocular staining score, *SGS* salivary gland scintigraphy, *SWS* stimulated whole saliva, *TBUT* tear break-up time, *UWS* unstimulated whole saliva

**Table 5:** Matrix of subjective oral and ocular assessments in the included studies, among which correlation and/or association analysis was performed. Bolded reference indicates at least one significant correlation between the assessments (sometimes multiple correlations were carried out, for example due to different patient groups). For non-significant results, only the available numerical values were included in this table

Ocular assessments Oral assessments	OSDI		MDEQ		Self-reported symptoms/questionnaires	
	*N* studies with significant results/total *N*	Ref.	*N* studies with significant results/total *N*	Ref.	*N* studies with significant results/total *N*	Ref.
S-XI	1/1	[61]	1/1	[61]	1/1	[75]
presence/diagnosis of xerostomia symptoms	1/1	[61]	1/1	[61]	3/3	[57, 69, 75]
self-reported symptoms/questionnaires	0/1	[66]	NR		10/10	[53–55, 64, 66, 69, 71, 74, 76, 77]

Abbreviations: *MDEQ* McMonnies Dry Eye Questionnaire, *NR* no statistics for associations and/or correlations reported, *OSDI* Ocular Surface Disease Index, *S-XI* Summated Xerostomia Inventory

### Data extraction

The data synthesis was executed based on the type of oral and ocular assessments, between either objective or subjective measurements only (Tables 4 and 5) or between both (Table S5). Correlation and/or association between objective only (*n* = 29), subjective only (*n* = 19) and between objective and subjective (*n* = 42) oral and ocular assessments was determined.

### Type of assessment

#### Objective assessments

Significant correlation and/or association between objective oral and ocular assessments was found in 10 out of 29 studies (Table 4). In all the studies that assessed the relationship between objective assessments, there were 2597 participants: case-control studies (*n* = 1075 patients and *n* = 695 controls), cross-sectional studies (*n* = 799), and observational cohort studies (*n* = 28)

Two studies [51, 67] observed a significant correlation between UWS and Schirmer I test results. On the other hand, no correlation or association between these assessments was reported in further three studies [59, 60, 61]. No correlation and/or association was found between UWS and ocular staining score (OSS) [59, 61, 66], presence/diagnosis of KCS [59]. In contrast, there was a correlation between UWS and van Bijsterveld score [51].

Correlation between SWS and Schirmer I test was found in two out of four cohorts [61, 69]. No correlation and/or association was found between SWS and TBUT [61, 66]. Ture et al. [66]. , but not Hynne et al. [61]. recorded correlation between SWS and OSS. Wangkaew et al. [69] reported correlation between SWS and KCS in two out of three patient groups.

Both parotid [67], and submandibular/sublingual flow rate [52] were correlated with Schirmer I test. Another study assessed correlation between MSG flow rate and Schirmer I test, but there was no significance [70].

In one study, MSG biopsy focus score correlated with OSS, but not TBUT [66]. On the other hand, SGS results were not correlated with Schirmer I test in two studies [65, 72]. SGS parameters were correlated with OSS, but not TBUT [66].

#### Subjective assessments

On the other hand, significant correlation and/or association between subjective oral and ocular assessment was found in 18 out of 19 studies (Table 5). In the studies that assessed the relationship between subjective assessments there were 5751 participants: case-control studies (*n* = 300 patients and *n* = 229 controls), cross-sectional studies (*n* = 5086), and observational cohort studies (*n* = 136).

There was correlation between S-XI and OSDI, MDEQ [61], and also dry eyes symptoms frequency [75]. XF and XF > 3 results correlated with MDEQ, but only XF and not XF > 3 correlated with OSDI [61]. Billings et al. [57] reported an association between xerostomia and self-reported dry eyes in females and males. In line with this, presence of symptomatic xerostomia and self-reported dry eye complaints frequency were positively correlated [75]. Furthermore, there was a correlation between presence/diagnosis of xerostomia symptoms and self-reported dry eyes in two out of three patient groups [69].

All 10 studies that assessed the correlation and/or association between self-reported dry mouth and self-reported dry eyes were significant. Three studies found self-reported dry mouth a predictive risk factor for self-reported dry eyes [55, 74, 76]. On the other hand, in two studies, self-reported dry eyes were a risk factor for self-reported dry mouth [64, 77]. Three studies recorded a correlation between self-reported dry mouth and dry eyes [53, 54, 69], except one patient group [69]. Furthermore, there was a correlation between the number of self-reported complaints of dry mouth and those of dry eyes [71]. Oral-visual analogue scale (VAS) results were positively correlated with ocular-VAS [66].

#### Subjective versus objective assessments

It is also worth highlighting some of the correlations and/or association that were assessed between objective and subjective oral and ocular measurements, where significance was found in 15 out of 42 studies (Table S5). In the studies that assessed the correlation and/or association between subjective and objective assessments, there was 2930 participants: case-control studies (*n* = 316 patients and *n* = 296 controls), cross-sectional studies (*n* = 2059), and observational cohort studies (*n* = 259).

Firstly, Lackner et al. [62] found correlation between UWS and OSDI, despite opposite results reported in two studies [54, 61]. On the other hand, there was a correlation between presence/diagnosis of xerostomia symptoms and Schirmer I test in two studies [61, 69]. However, Rasker et al. [71] found correlation between self-reported dry mouth and Schirmer I test. In line with this, Wangkaew et al. [69] reported correlation between self-reported dry mouth and Schirmer I test, but in contrary, Lackner et al. [62] did not find this relationship. Furthermore, oral sensitivity, which takes into consideration objective and subjective assessments, was correlated with ocular sensitivity (objective and subjective assessments) [56].

### Cohort type

#### General population

Four studies examined the correlation and/or association between dry mouth and dry eyes complaints in general population, in these cases individuals attending local dental clinics and/or ophthalmological clinics [55, 57, 67, 77]. There were three cross-sectional (*n* = 2738) and one case-control study (*n* = 486 patients and *n* = 156 controls).

In Polish patients attending either dental or optometrist clinics, there was a positive correlation between both UWS and parotid flow and Schirmer I test in both healthy controls and patient groups [67].

Regarding associations between subjective assessments, the association between xerostomia and self-reported dry eyes was found in both females and males in USA [57]. Additionally, Bergdahl [55], reported that Swedish dental clinic attendees with self-reported dry mouth complaints had higher odds ratio of self-reported dry eye complaints, whilst Villa and Abati [77] found that self-reported dry eyes were a risk factor for self-reported dry mouth in Italian population.

These findings suggest that there is an association between sicca symptoms in general population.

#### Elderly population

Correlation and/or association between ocular and oral sicca complaints in elderly population was assessed by four cross-sectional studies (*n* = 2070) [61, 64, 75, 76].

In the Norwegian cohort [61], UWS and SWS did not correlate with results of any of the objective ocular assessments – OSS, Schirmer I test, and TBUT.

In the Danish population [64], individuals who had self-reported ocular dryness had almost five times the odds of also having self-reported oral dryness. Furthermore, self-reported feeling of dry mouth was a risk for self-reported dry eyes in elderly Thais [69]. In Australian population [75], there was a positive correlation between both S-XI scores and also the presence of symptomatic xerostomia and self-reported dry eye symptoms frequency. In Norwegian patients [61], there was a weak positive correlation between S-XI and both MDEQ and OSDI. Furthermore, S-XI > 10 was correlated with MDEQ > 10.5 and with OSDI > 12. In case of XF, XF and OSDI were weakly positively correlated, XF and MDEQ were moderately positively correlated, while XF > 3 was correlated with MDEQ > 10.5, but not OSDI > 12.

In the Danish cohort, there was no association found between salivary flow assessments and self-reported dry eye [64]. In Norwegian elderly [61], UWS and SWS did not correlate with OSDI and MDEQ. Furthermore, there was no correlation between S-XI > 10 and any objective dry eye assessments. XF > 3 was correlated with Schirmer I test ≤ 10 mm/5 min, and OSS ≥ 1, but not TBUT ≤ 10 s.

In elderly cohort, majority of significant associations were between subjective dry mouth and dry eyes assessments.

#### Sjögren’s disease

Sicca complaints are one of the most common manifestations in SjD patients. In the five studies that either did not distinguish between pSjD and aSjD patients or grouped them as one cohort for statistical analysis [52, 65, 68, 70, 72], there were three case-control (*n* = 347 patients, *n* = 348 controls) and two were cross-sectional (*n* = 222) studies.

SWS of submandibular/sublingual salivary glands was correlated with Schirmer I test [52]. On the other hand, Chiu et al. [72] found no association between SGS and Schirmer I test. However, Xin et al. [68] reported correlations between both UWS and SWS and SSI ocular domain score.

Further eight studies assessed the associations in pSjD patients only [51, 56, 58, 59, 62, 63, 66, 73]. There was one case-control (*n* = 16 patients and *n* = 27 controls), two observational (*n* = 151) and five cross-sectional (*n* = 2341) studies.

There was a correlation between UWS and Schirmer I test, and UWS and van Bijsterveld score [51]. By contrast, Ture et al. [66] found no correlation between UWS and OSS and UWS and TBUT. Likewise, Da Cunha et al. [59]. found no correlation between abnormal UWS and any objective ocular test results. SWS results were correlated with OSS, but not TBUT [66]. Ture et al. [66] further reported a correlation between OSS and SGS parameters and MSG biopsy focus score. Yet, there was no correlation between SGS parameters, MSG biopsy results and TBUT results [66]. Furthermore, SGS parameters did not correlate with Schirmer I test [65].

On the contrary, subjective dry mouth assessments, such as oral-VAS results were positively correlated with ocular-VAS, but oral-VAS results were not correlated with OSDI [66].

Further, there was a negative correlation between UWS and OSDI [62]. Likewise, UWS had significant inverse correlation to the number of self-reported dry eye complaints [63], and there was a significant correlation between UWS and self-reported dry eyes [58]. Lackner et al. and Ture et al. found no correlation between UWS and VAS-sicca-eyes [62], and UWS and ocular-VAS [66]. SWS and OSDI were correlated [62], but opposite to that, SWS results did not correlate with subjective dry eye assessments [62, 66]. LSG biopsy [73], MSG biopsy and SGS results [66] did not correlate with subjective dry eye assessments either. As well, Lackner et al. [62] described no correlation between neither XI nor VAS-sicca-mouth and Schirmer I test. There was no correlation between oral-VAS and neither OSS nor TBUT [66]. In contrast, Bezzina et al. [56] observed that oral and ocular sensitivity (which take into consideration both objective and subjective symptoms) were correlated.

In summary, in SjD patients there was only one assessment carried out between subjective dry mouth and dry eyes assessments when compared to multiple within other cohort groups. Additionally, there were mixed outcomes in the associations carried out between objective only and between objective and subjective assessments.

#### Other systemic diseases

Four studies focused on systemic diseases, such as systemic lupus erythematosus (SLE) [60, 69], RA [60, 69], systemic sclerosis (SCL) [69, 71], and Stevens–Johnson syndrome (SJS) [70]. All four studies were case-control studies (*n* = 272 patients and *n* = 421 controls)

No correlation was reported between MSG flow rates and Schirmer I test in SJS patients [70]. There was no correlation observed between neither UWS [60] nor SWS [69] and Schirmer I test in SLE patients. Furthermore, Wangkaew et al. [69] found no correlation between SWS and KCS diagnosis in individuals with SLE. On the other hand, in RA patients, there was a correlation between SWS results and Schirmer I test [69], but not between SWS and presence of KCS symptoms [69]. In SCL patients, there was no correlation between SWS and Schirmer I test, but SWS and presence of KCS symptoms were significantly correlated [69].

In SCL patients the total number of self-reported complaints of dry mouth and those of dry eyes was correlated [71]. In both RA and SLE patients, there was a correlation between self-reported dry mouth and self-reported dry eyes, and xerostomia and self-reported dry eyes [69]. In contrast, in SCL patients, there was no correlation between self-reported dry mouth or xerostomia and self-reported dry eyes [69].

In SLE patients [69], there was a correlation between self-reported dry mouth and Schirmer I test, and xerostomia and Schirmer I test. Furthermore, there was a correlation between self-reported dry mouth and KCS, and xerostomia and KCS. However, there was no correlation between SWS and self-reported dry eyes. In RA patients, the only correlation between objective and subjective assessments recorded was between self-reported dry mouth or xerostomia and KCS [69]. Wangkaew et al. [69] found no associations between objective and subjective assessments in SCL patients, yet Rasker et al. [71] recorded a correlation between self-reported dry mouth and Schirmer I test.

In these patient cohorts, there is no apparent trend indicating associations between only objective, or objective and subjective assessments.

#### Cancers and transplants

Three studies examined sicca symptoms in cancer and transplant patients [53, 54, 74]. There was one cross-sectional (*n* = 212), one observational cohort (*n* = 136) and one case-control study (*n* = 124 patients and *n* = 10 controls).

No associations between objective assessment, such as UWS or SWS results and OSDI were found in differentiated thyroid carcinoma (DTC) patients [54].

In both chronic graft versus host disease (cGVHD) [53] and DTC [54] patients, there was a correlation between self-reported dry mouth and self-reported dry eyes. Alike, in patients who undergone allogeneic hematopoietic progenitor cell transplantation (HPCT), self-reported dry mouth was a risk and predictive factor for self-reported dry eyes [74]. Furthermore, there was a correlation between self-reported dry mouth and OSDI in DTC patients [54].

No associations were reported between salivary flow measurements and OSDI. On the other hand, there was a correlation between self-reported dry mouth and OSDI [54]. Alike, in HPCT patients, LSG biopsy was a risk and predictive factor for self-reported dry eyes [74].

In all three cancer/transplant cohorts, there was association between self-reported subjective sicca complaints.

## Discussion

The association between dry mouth and dry eyes was systematically reviewed based on the currently available scientific literature. The most evident observation can be made about the associations between subjective dry mouth and dry eyes. 95% of the included studies found significance between subjective assessments, and within that, all studies that analysed the correlation and/or association between the subjective self-reported complaints/questionnaires were statistically significant, with the exception of one group of SCL patients [69]. On the other hand, fewer correlations and/or associations were found between objective measurements of dry mouth and dry eyes. For example, the association between UWS and Schirmer I test was found in two out of five studies [51, 59, 60, 61, 67], which is unanticipated, as these methodologies are considered gold standard for sicca assessment. Significant findings were neither evident between objective or subjective oral and ocular assessments, for example subjective self-reported dry mouth and Schirmer I test results (two out of three) [62, 69, 71] or UWS and subjective self-reported dry eyes (two out of six) [54, 58, 62, 63, 64, 66]. These results are thought-provoking, as strong association between objective oral and ocular assessments was expected, in contrary to the association between subjective symptoms [84, 85].

It is difficult to draw clear conclusions based on these findings, due to the variety of assessment methods applied. The extracted data shows high level of heterogeneity, as various methodology was applied and compared, e.g. some studies investigated correlation and/or association only between UWS and Schirmer I test, and others used only self-reported questions to assess dry mouth and dry eyes. Furthermore, there was a variability in the applied methodology, for example in the way of executing UWS measurements. Depending on the study design, UWS collection was carried out over five, 10–15 min. This could influence the final data used for oral and ocular association assessments.

The matters mentioned above visualize the difficulty with standardisation of the guidelines and protocols. The lack of appropriate guidelines can have clinical implications. First and foremost, the non-specific symptoms can be attributed to, for example aging or medications, leading to underdiagnosis. On the other hand, diseases such as SjD may be missed or diagnosed late (pSjD is often diagnosed using European League Against Rheumatism (EULAR) SS (SjD) Patient Reported Index (ESSPRI) and the EULAR SS (SjD) Disease Activity Index (ESSDAI) [86], lip/salivary gland biopsies and blood tests for specific autoantibodies – e.g. anti-Ro/SSA, anti-La/SSB, rheumatoid factor (RF) [87]), delaying systemic care and increasing the risk of complications (e.g. development of lymphoproliferative disorders [88, 89], dental caries, corneal ulcers). With increased average life expectancy, more elderly will be affected by dry mouth and dry eyes, for example due to polypharmacy, hence more emphasis needs to be put on the research on the mechanisms behind those symptoms. Next matter involves the variability in the use of criteria and tools by the clinicians (e.g. non-validated questionnaires, use of various cut-off for hyposalivation [90, 91]), leading to diagnostic inconsistency between providers, which later has impact on long-term monitoring of disease progression and appropriate referrals to specialists. Appropriate and timely diagnosis also requires co-operation between different health professionals, as patients with sicca symptoms often first approach general practitioners (GPs), ophthalmologists or dentists with their symptoms, and only later are referred to rheumatologists and other specialists. GPs and dentists often do not fully understand the burden of sicca symptoms [92] or dismiss them when patients have other, more pressing health issues. Without clear protocols, treatment may be based on the symptom severity rather than the underlying cause. This increases the risk of patients receiving unnecessary or ineffective therapies. Inaccurate diagnosis and treatment can contribute to patient’s already reduced quality of life and deepen the stress and frustration around experiencing the discomfort due to dryness. Lastly, repeated referrals and diagnostic testing due to the uncertainty of the underlying causes can increase healthcare costs.

Some of the studies included in this review had limitations. For example, some of the methodologies applied to assess oral and ocular dryness are not commonly applied in clinics or are novel, such as the use of modified Schirmer I test [67] or MSG count [70]. It is also worth noting that some studies used self-reported non-validated questions for the diagnosis of xerostomia or dry eyes [58, 77]. As well as, some studies put the emphasis either on oral or ocular assessments and did not investigate the other in depth, e.g. measure UWS, SWS and subjective dry mouth complaints with a questionnaire, but only used one self-reported question to assess dry eye complaints [55]. In general, the overall quality of studies was primarily graded low according to the NIH study quality assessment tools, with only one study scoring “good” [54]. Furthermore, just two studies carried out sample size calculation to establish the power of their studies [54, 68]. Finally, there was a lack of consistency in the disease nomenclature, for example the term “xerostomia” was used to describe both subjective and objective measurements. Nowadays, the term should be exclusively applied to the subjective feeling of dry mouth. This review provides a general and broad outline – various methodologies and assessments were allowed within the eligibility criteria, which resulted in high heterogeneity of effect measures, and consequently caused more difficulty in forming conclusions. Moreover, due to the heterogeneity of the outcomes, meta-analysis could not be performed. Lastly, the analysed literature did not provide deeper insight into understanding the causes and mechanisms of sicca symptoms.

## Conclusions

This systematic review is a steppingstone in the understanding of the correlation and/or association between dry mouth and dry eyes and their underlying mechanisms. From this research, we can conclude that there is a need for better standardisation and protocols for the diagnosis of sicca symptoms. Lack of standardised diagnostic guidelines for dry mouth and dry eyes can cause delays in appropriate care, increase healthcare costs, and negatively affect patient outcomes. Developing standardized, evidence-based diagnostic criteria would greatly enhance early recognition, treatment, and overall patient management. It also emphasizes the need for co-operation between general and specialised healthcare professionals, and fundamental researchers. However, these issues are not highlighted in the currently available literature

The findings of this review are not exhaustive, while they suggest association between subjective dry mouth and dry eyes, more clinical data is necessary for clear conclusions. In addition, an in-depth analysis of relationship between types of assessment (e.g. salivary flow and Schirmer I test) and specific cohorts (e.g. elderly, SjD patients) should be considered for a better understanding of the matter.

In the future, this will allow for better diagnostics and factoring predictions for patients experiencing sicca symptoms.

## Supplementary Information

Below is the link to the electronic supplementary material.

Supplementary Material 1
