# Supplementary material for: Association between dry mouth and dry eyes: a systematic literature review of clinical evidence
Source: Rheumatol Int. 2025 Aug 28;45(9):209. doi: 10.1007/s00296-025-05958-5 (PMC12394332; doi:10.1007/s00296-025-05958-5)
Supplement: Supplementary file 1 — Supplementary Material 1 [file 296_2025_5958_MOESM1_ESM.docx]

Supplementary data

**Table S1.** Search terms applied in the search strategy.

| Database | Search query | Filters |
| --- | --- | --- |
| PubMed | ("dry eyes" OR "ocular dryness" OR "eyes dry*" OR "dry eyes disease" OR "dry eyes syndrome" OR "keratoconjunctivitis sicca" OR "dysfunctional tear syndrome" OR "lacrimal keratoconjunctivitis" OR "xerophthalmia") AND ("dry mouth" OR "oral dry*" OR "mouth dry*" OR "xerostomia" OR "hyposaliv*" OR "saliva* flow") AND ("association" OR "correlat*" OR "relationship") | "Humans" and "English" |
| Web of Science Core Collection | (ALL=(dry eyes) OR ALL=(ocular dryness) OR ALL=(eyes dry* ) OR ALL=(dry eyes disease) OR ALL=(dry eyes syndrome) OR ALL=(keratoconjunctivitis sicca) OR ALL=(dysfunctional tear syndrome) OR ALL=(lacrimal keratoconjunctivitis) OR ALL=(xerophthalmia)) AND (ALL=(dry mouth) OR ALL=(oral dry*) OR ALL=(mouth dry*) OR ALL=(xerostomia) OR ALL=(hyposaliv*) OR ALL=(saliva* flow)) AND (ALL=(association) OR ALL=(correlat*) OR ALL=(relationship)) | "English" |

**Table S2.** Results of oral and ocular assessments. Percentages and n-values were calculated where missing. Bolded p-values indicate significance. Values are reported as mean ± SD unless specified.

| First author, publication year | Country of study population | Study type | Population | Participant characteristics  [total (n), female/male (n), age (y)] | Oral and ocular assessments carried out  [subjective; objective; objective & subjective] | Results of oral and ocular assessments  [subjective; objective; objective & subjective] |
| --- | --- | --- | --- | --- | --- | --- |
| Bassim, *et al*. [53], 2015 | USA | Cross-sectional | cGVHD patients | n=212, F 99/M 113  (median ± range) 48 ± 18-70 y | **Oral:** self-reported (0-10 scale);  UWS (5 min) (n=112)  **Ocular:** self-reported (0-10 scale);  Schirmer I test (5 min) | **Oral:** 11% (n=23) UWS ≤1 mL/5 min.  **Ocular:** 62.7% (n=133) Schirmer I test ≤5 mL/5 min**.** |
| Baudin, *et al.* [54], 2023 | France | Observational cohort | DTC patients undergone thyroidectomy, receiving ^131^I-therapy with ^131^I-activity of 1.1 GBq or 3.7 GBq | n=136, F 97/M 39  47.1 ± 14.1 y   - n=44, F/M NR   NR y   - n=92, F/M NR   NR y | **Oral:** self-reported;  UWS (5 min), SWS (5 min)  **Ocular:** self-reported, OSDI | **Oral:** UWS 0.6 ± 0.4 mL/min in 1.1 GBq and 0.8 ± 0.5 mL/min in 3.7 GBq patients. SWS 1.9 ± 0.6 mL/min in 1.1 GBq and 2.2 ± 1.0 mL/min in 3.7 GBq patients.  **Ocular:** OSDI score 8.0 ± 12.0 in 1.1 GBq and 6.0 ± 9.5 in 3.7 GBq patients. |
| Bergdahl [55], 2000 | Sweden | Cross-sectional | Dental clinic attendees | n=1427, F 758/M 669  10 groups in the range 20-69 y | **Oral:** self-reported;  UWS (10 min), SWS (3 min)  **Ocular:** self-reported | **Oral:** 22% (n=314) “yes” to “Does your mouth usually feel dry?”; 2.3% (n=33) taste disturbances; 4.7% (n=67) burning mouth.  UWS in F: 0.26 ± 0.21 mL/min and in M: 0.33 ± 0.26 mL/min; SWS in F: 2.02 ± 0.93 mL/min and in M: 2.50 ± 1.06 mL/min; 2.2% (n=32) no UWS; 0.1% (n=1) no SWS.  **Ocular:** 9.5% (n=136) subjective dry eye complaints. |
| Bezzina, *et al.* [56], 2017 | UK | Cross-sectional | pSjD patients | n=688, F 651/M 37  58 ± 12.5 y | **Oral:** self-reported (0-10 scale) (n=681);  UWS (15 min);  oral sensitivity (-5-+5 scale) (n=681)  **Ocular:** self-reported (0-10 scale) (n=681);  Schirmer I test (5 min) (n=671);  ocular sensitivity (-5-+5 scale) (n=681) | **Oral:** Subjective dry mouth complaints rated 6.0 ± 2.9.  UWS 0.9 ± 1.9 mL/15 min.  Oral sensitivity -1.24 ± 1.6.  **Ocular:** Subjective dry eye complaints 5.6 ± 2.8.  Schirmer I test 6.2 ± 7.6 mm/5 min.  Ocular sensitivity -0.42 ± 2.2. |
| Billings, *et al.* [57], 1996 | USA | Cross-sectional | Community-dwelling adults | n=710, F 484/M 226  range 19-88 y | **Oral:** self-reported;  UWS (2 min), SWS (1 min)  **Ocular:** self-reported | **Oral:** 22.1% (n=157) (F: 24% (n=116); M: 18.1% (n=41) subjective dry mouth; 6.1% (n=43) (F: 6.8% (n=33), M: 4.4% (n=10)) a feeling of too little saliva; 10.8% (n=77) (F: 12.6% (n=61), M: 7.1% (n=16)) problems with dry food; 7.5% (n=53) (F: 8.7% (n=42), M: 4.9% (n=11)) cracked lips; 4.1% (n=29) (F: 4.3% (n=21), M: 3.5% (n=8)) difficulty swallowing.  UWS in F with subjective dry mouth: <30 y 0.17 ± 0.17 mL/min, 30-49 y 0.15 ± 0.15 mL/min, 50-69 y, 0.16 ± 0.17 mL/min and <70 y 0.11 ± 0.12 mL/min. UWS in F without subjective dry mouth: <30 y 0.26 ± 0.19 mL/min, 30-49 y 0.22 ± 0.15 mL/min, 50-69 y 0.18 ± 0.24 mL/min and <70 y 0.14 ± 0.13 mL/min. UWS in M with subjective dry mouth: <30 y 0.24 ± 0.20 mL/min, 30-49 y 0.14 ± 0.15 mL/min, 50-69 y 0.09 ± 0.06 mL/min and <70 y 0.18 ± 0.13 mL/min. UWS in M without subjective dry mouth: <30 y 0.28 ± 0.28 mL/min, 30-49 y 0.29 ± 0.22 mL/min, 50-69 y 0.22 ± 0.15 mL/min and <70 y 0.20 ± 0.25 mL/min.  SWS in F with subjective dry mouth: <30 y 1.91 ± 1.06 mL/min, 30-49 y 2.06 ± 1.40 mL/min, 50-69 y 1.81 ± 1.18 mL/min and <70 y 1.07 ± 0.86 mL/min. SWS in F without subjective dry mouth: <30 y 2.56 ± 1.38 mL/min, 30-49 y 2.30 ± 1.08 mL/min, 50-69 y 1.74 ± 1.05 mL/min and <70 y 1.31 ± 0.98 mL/min. SWS in M with subjective dry mouth: <30 y 2.07 ± 1.10 mL/min, 30-49 y 1.58 ± 0.88 mL/min, 50-69 y 1.61 ± 1.38 mL/min and <70 y 1.74 ± 1.54 mL/min. SWS in M without subjective dry mouth: <30 y 2.50 ± 1.45 mL/min, 30-49 y 2.04 ± 1.34 mL/min, 50-69 y 1.82 ± 1.32 mL/min and <70 y 1.61 ± 1.50 mL/min.  **Ocular:** 12.5% (n=89) subjective dry eye; 5.6% (n=40) with subjective dry mouth and subjective dry eye. |
| Błochowiak [58], 2024 | Poland | Cross-sectional | SjD patients | n=50, F 43/M 7  52 ± 10.9 y | **Oral:** self-reported (xerostomia symptoms);  LSG biopsy, UWS (15 min)  **Ocular:** self-reported;  Schirmer I test (5 min) | **Oral:** 62% (n=31) subjective dry mouth;4% (n=2) salivary gland inflammation; 46% (n=23) UWS ≤1.5 mL/15 min; UWS (median ± IQR) 2.0 ± 2.5 mL/15 min; patients with xerostomia UWS 1.0 ± 2.5 mL/min; patients without xerostomia UWS 3.0 ± 3.0 mL/min.  **Ocular:** 62% (n=31) subjective dry eye; 77.4% (n=24) with xerostomia and 36.8% (n=7) SjD patients without xerostomia subjective dry eye; 70% (n=35) Schirmer I test ≤5 mm/5 min; Schirmer I test (median ± IQR) 5.0 ± 7.0 mm/5 min right eye and 5.0 ± 7.0 mm/5 min left eye; 71% (n=22) with xerostomia Schirmer I test ≤5 mm/5 min; 68.4% (n=13) without xerostomia Schirmer I test ≤5 mm/5 min. |
| Chiu, *et al.* [72], 2020 | Taiwan | Case-control | SjD patients and non-SjD sicca patients | - n=247, F 219/M 28 56.6 ± 14.7 y - n=268, F 221/M 47 54.7 ± 15 y | **Oral:** LSG biopsy, SGS  **Ocular:** Schirmer I test (n=421) | **Oral:** SGS delay “markedly” in 45.3% (n=112) SjD vs. 35.5% (n=95) non-SjD, “moderately” in 41.3% (n=102) SjD vs. 44.8% (n=120) non-SjD, “mildly” in 11% (n=27) SjD vs. 12% (n=31) non-SjD and “negative” in 2% (n=6) SjD vs. 8% (n=22) non-SjD; 13.6% (n=70) patients positive LSG.  **Ocular:** Schirmer I test “positive” 52.8% (n=124) SjD vs. 32.3% (n=60) non-SjD, “equivocal” 35.3% (n=83) SjD vs. 40.9% (n=76) non-SjD, and “negative” 12% (n=28) SjD vs. 27% (n=50) non-SjD. |
| Da Cunha, *et al.* [59], 2022 | France | Cross-sectional | pSjD patients and non-SjD sicca patients**;** later divided into “severe KCS” and “non-severe KCS” | - n=253, F 245/M 8   56.6 ± 13 y   - n=108, F 98/M 10   56.4 ± 13.8 y;  divided into:   - n=94, F 90/M 4   58 ± 13.5 y   - n=159, F 155/M 4 - 55.9 ± 14.1 y | **Oral**: UWS (NR min), MSG biopsy  **Ocular:** self-reported (DED-VAS, 0-10);  fluorescein staining and lissamine green staining (graded as OSS), Schirmer I test (5 min), TBUT | **Oral:** 46.2% (n=104) of pSjD UWS <0.1 mL/min, and UWS 0.2 ± 0.1 mL/min; 57.9% (n=44) severe KCS patients UWS <0.1 mL/min; 42.2% (n=57) non-severe KCS patients UWS <0.1 mL/min.  **Ocular**: DED-VAS 5.6 ± 3.1 in pSjD, and 5.8 ± 2.9 in non-SjD; 53.3% (n=135) pSjD Schirmer I test ≤5 mm/5 min, and Schirmer I test 7.8 ± 7.6 mm/5 min; 20.2% (n=22) non-SjD Schirmer I test ≤5 mm/5 min, and Schirmer I test result 15.7 ± 10.6 mm/5 min; 95.2% (n=241) pSjD TBUT ≤10 s, and TBUT 4.3 ± 3.1 s. 89.8% (n=97) non-SjD TBUT ≤10 s, and TBUT 4.4 ± 2.6 s; in pSjD, corneal OSS 1.6 ± 0.8, and conjunctival OSS 3.1 ± 1.8; 54.9% (n=139) pSjD OSS ≥5, and OSS 4.8 ± 2.5; in non-SjD, corneal OSS 0.9 ± 0.6, and conjunctival OSS 1.6 ± 1.6. 15.7% (n=17) non-SjD total OSS ≥5, and OSS 2.5 ± 2.0. |
| Fernandez Castro, *et al.* [73], 2018 | Spain | Cross-sectional | pSjD patients with not severe dry eyes symptoms and severe/very severe dry eyes symptoms | - n=59, F 57/M 2   49.3 ± 13.9 y   - n=378, F 359/M 19 - 50.6 ± 12.7 y | **Oral:** LSG biopsy (n=193)  **Ocular:** self-reported;  fluorescein staining (n=81), lissamine green staining (n=19), Rose Bengal staining (n=144), Schirmer I test (5 min) (n=402) | **Oral:** 44.1% (n=26) of not severe dry eye and 28.3% (n=107) of severe/very severe dry eye patients LSG biopsy focus score ≥ 1.  **Ocular:** 94.0% (n=411) complained of daily, persistent, troublesome dry eye; 92.0% (n=402) sensation of sand in their eyes; 92.0% (n=371) pathological Schirmer I test results. |
| Gilboe, *et al.* [60], 2001 | Norway | Case-control | Patients with SLE or RA with sicca symptoms and suspicion of aSjD, and healthy controls | - n=81, F 72/M 9   (mean ± range) 44 ± 20-70 y   - n=81, F 72/M/9   44 ± 22-69 y   - n=81, F 72/M 9 - 44 ± 20-70 y | **Oral:** self-reported;  UWS (15 min)  **Ocular:** self-reported;  Schirmer I test (15 min) | **Oral:** 37% (n=30) of SLE, 30% (n=24) of RA, and 9% (n=7) of controls responded “yes” to “Dry mouth daily for more than 3 months?”. 11% (n=9) of SLE, 6% (n=5) of RA, and 1% (n=1) of controls responded “yes” to “Experienced swollen salivary glands?”. 47% (n=38) of SLE, 34% (n=28) of RA, and 19% (n=15) of controls responded “yes” to “Drink liquids to swallow dry food?”. 52% (n=42) of SLE, 42% (n=34) of RA, and 21% (n=17) of controls symptoms at least of dry eye; UWS 4.30 ± 3.54 mL/15 min in SLE and 4.46 ± 3.12 mL/15 min in RA.  **Ocular:** 28% (n=23) of SLE, 14% (n=11) of RA, and 10% (n=8) of controls responded “yes” to “Dry eyes daily for more than 3 months?”. 41% (n=33) of SLE, 24% (n=19) of RA, and 13% (n=11) of controls responded “yes” to “Sensation of sand or gravel?”. 9% (n=7) of SLE, 1% (n=1) of RA, and 2% (n=2) of controls responded “yes” to “Use of tear substitutes more than 3 times a day?”. 43% (n=35) of SLE, 27% (n=22) of RA, and 16% (n=13) of controls had at least of dry eye symptom.  Schirmer I test of the right eye 14.4 ± 13.1 mm/15 min in SLE, and 18.9 ± 13.3 mm/15 min in RA;Schirmer test of the left eye 15.4 ± 13.1 mm/15 min in SLE, and 22.9 ± 12.4 mm/15 min in RA. |
| Hynne, *et al.* [61], 2022 | Norway | Cross-sectional | 65-year-old population in Oslo | n=150, F 82/M 65  65 ± 0 y | **Oral:** CODS, S-XI, XF;  UWS (5 min), SWS (5 min) (n=148)  **Ocular:** MDEQ (n=148), OSDI (n=149);  fluorescein staining (graded as OSS), Schirmer I test (5 min) (n=146), TBUT (n=139) | **Oral**: XF 1.6 ± 0.7; S-XI score 6.7 ± 1.7;CODS 2.0 ± 1.3; 3.3% (n=5) S-XI (>10). 8% (n=12) XF ≥3; UWS 0.4 ± 0.3 mL/min; SWS 1.9 ± 0.9 mL/min; 8% (n=12) UWS ≤0.1 mL/min and 4.7% (n=7) SWS ≤0.7 mL/min.  **Ocular:** MDEQ 6.3 ± 4.0; 16% (n=24) MDEQ >10.5; OSDI 8.3 ± 11.3. 27.3% (n=41) OSDI >12; Schirmer I test 12.4 ± 8.6 mm/5 min; 48.7% (n=73) Schirmer I test ≤10 mm/5 min; TBUT 9.0 ± 6.2 s; 64% (n=96) TBUT ≤10 s, and 39.3% (n=59) TBUT ≤5 s; OSS 0.8 ± 1.2; 24.3% (n=68) OSS ≥1. |
| Kalk, *et al.* [52], 2002 | the Netherlands | Cross-sectional | pSjD, aSjD and non-SjD patients | - n=32, F 30/M 2   53 ± 14 y   - n=25, F 19/M 6   58 ± 14 y   - n=23, F 21/M 2 - 48 ± 12 y | **Oral:** self-reported;  submandibular/sublingual unstimulated flow rate (5 min), submandibular/sublingual stimulated flow rate (10 min), parotid unstimulated flow rate (5 min) (n=28), parotid stimulated flow rate (10 min), UWS (15 min), SWS (15 min)  **Ocular:** self-reported;  Rose Bengal staining, Schirmer I test (5 min), TBUT | **Oral:** 87% (n=28) pSjD, 92% (n=23) aSjD and 78% (n=18) non-SjD had subjective dry mouth; 94% (n=30) pSjD, 96% (n=24) aSjD and 0% non-SjD positive gland biopsy; 97% (n=31) pSjD, 92% (n=23) aSjD and 61% (n=14) non-SjD positive oral test(s); 100% (n=28) pSjD, 76% (n=16) aSjD and 8% (n=3) non-SjD silactasia present (parotid flow rate); 72% (n=23) pSjD, 83% (n=19) aSjD and 57% (n=13) non-SjD UWS ≤1.5 mL/15 min; UWS 0.11 ± 0.18 mL/15 min for pSjD, 0.05 ± 0.08 mL/15 min for aSjD and 0.16 ± 0.22 mL/15 min for non-SjD; parotid unstimulated flow rate 0.02 ± 0.03 mL/min/gland for pSjD, 0.01 ± 0.03 mL/min/gland for aSjD and 0.03 ± 0.07 mL/min/gland for non-SjD; submandibular/sublingual unstimulated flow rate 0.07 ± 0.12 ml/min/SM/SL gland for pSjD, 0.02 ± 0.03 ml/min/SM/SL gland for aSjD and 0.10 ± 0.11 ml/min/SM/SL gland for non-SjD; SWS 0.25 ± 0.30 mL/10 min for pSjD, 0.55 ± 0.68 mL/10 min for aSjD and 0.79 ± 0.44 mL/10 min for non-SjD; parotid stimulated flow rate 0.13 ± 0.15 mL/min/gland for pSjD, 0.15 ± 0.19 mL/min/gland for aSjD and 0.19 ± 0.12 mL/min/gland for non-SjD; submandibular/sublingual stimulated flow rate 0.25 ± 0.30 ml/min/SM/SL gland for pSjD, 0.24 ± 0.35 ml/min/SM/SL gland for aSjD and 0.42 ± 0.25 ml/min/SM/SL gland for non-SjD.  **Ocular:** 84% (n=27) pSjD, 92% (n=23) aSjD and 74% (n=17) non-SjD subjective dry eye; 71% (n=22) pSjD, 96% (n=24) aSjD and 48% (n=11) non-SjD positive eye test(s); 50% (n=16) pSjD, 64% (n=17) aSjD and 43% (n=10) non-SjD Schirmer I test ≤5 mm/min. 63% (n=19) pSjD, 79% (n=19) aSjD and 26% (n=6) non-SjD had Rose Bengal score ≥4; Schirmer I test 8.2 ± 8.2 mm/5 min for pSjD, 4.5 ± 4.7 mm/5min for aSjD and 10.2 ± 8.8 mm/5 min for non-SjD; Rose Bengal score 5.0 ± 2.4 for pSjD, 5.7 ± 2.1 for aSjD and 2.7 ± 2.0 for non-SjD; TBUT 3.4 ± 3.9 s for pSjD, 2.7 ± 3.3 s for aSjD and 5.7 ± 3.6 s for non-SjD. |
| Kasetsuwan, *et al.* [76], 2012 | Thailand | Cross-sectional | Elderly Thai population | n=625, F 392/M 233  65.3 ± 7.5 y | **Oral:** self-reported  **Ocular:** self-reported | **Oral:** 24.8% (n=155) subjective dry mouth.  **Ocular:** Discomfort reported as “none” by 96.3% (n=585), “mild” by 2.6% (n=16), “moderate” by 2.4% (n=15) and “severe” by 1.4% (n=9); foreign body sensation reported as “none” by 89.4% (n=559), “mild” by 4.5% (n=28), “moderate” by 4.5.% (n=28) and “severe” by 1.6% (n=10); dryness reported as “none” by 95.8% (n=599), “mild” by 1.8% (n=11), “moderate” by 1.4% (n=9) and “severe” by 1.0% (n=6); tearing reported as “none” by 82.9% (n=518), “mild” by 7.2% (n=45), “moderate” by 8.3% (n=52) and “severe” by 1.6% (n=10); itching reported as “none” by 84.0% (n=525), “mild” by 7.2% (n=45), “moderate” by 6.9% (n=43) and “severe” by 1.9% (n=12); photophobia reported as “none” by 87.0% (n=544), “mild” by 4.0% (n=25), “moderate” by 6.7% (n=42) and “severe” by 2.2% (n=14); burning sensation reported as “none” by 89.3% (n=558), “mild” by 5.6% (n=35), “moderate” by 3.8% (n=24) and “severe” by 1.3% (n=8); blurred vision after prolonged reading reported as “none” by 67.4% (n=412), “mild” 11.2% (n=70), “moderate” by 10.9% (n=68) and “severe” by 10.6% (n=66); any severe symptom reported by 14.2% (n=89); any severe symptom except blurred vision after prolong reading reported by 7.2% (n=45). |
| Lackner, *et al.* [62], 2021 | Austria | Observational cohort | pSjD patients | n=123, F 113/M 10  60.1 ± 12.3 y | **Oral:** self-reported (VAS, 0-10 scale), XI;  UWS (5 min), SWS (2 min)  **Ocular (n=43):** self-reported (VAS, 0-10 scale), OSDI;  fluorescein staining, Marx-line staining, meibomian gland secretion quality, quality of meibomian gland secretion, Schirmer I test (5 min), TBUT | **Oral:** UWS (median ± range) 0.2 ± 0-5 mL/5 min; SWS (median ± range) 1.5 ± 0-4.5 g/2 min.  **Ocular:** OSDI (median ± range) 54.2 ± 12.5–97.7; Schirmer I test (median ± range) 1.5 ± 0-35.0 mm/5 min. |
| Leite, *et al.* [74], 2006 | Brazil | Case-control | Patients undergone allogeneic HPCT and healthy controls | - n=124, F 44/M 80   31.5 ± 12.1 y   - n=10, F/M NR - NR y | **Oral:** self-reported;  LSG biopsy  **Ocular (Patients, n=33 and healthy controls, n=10):** self-reported;  fluorescein staining, MG dysfunction, Rose Bengal staining, Schirmer I test (5 min), TBUT | **Oral:** 48.4% (n=60) inflammatory infiltrate in the salivary glands.  **Ocular:** 32.3% (n=40) dry eye symptoms; 57.6% (n=19) ocular discomfort; 42.4% (n=14) Rose Bengal score >5; 42.4% (n=14) MG dysfunction; 64.0% (n=21) TBUT ≤5 s; 30.3% (n=10) KCS; 30.3% (n=10) Schirmer I test <5 mm/5 min. |
| Oxholm, *et al*. [51], 1989 | Denmark | Observational cohort | pSjD patients | n=28, F 24/M 4  (mean ± range) 51 ± 32-71 y | **Oral:** UWS (15 min)  **Ocular:** Schirmer I test (5 min), TBUT, van Bijsterveld score | **Oral:** At first examination, UWS 2.50 ± 3.81 mL/15 min, at second 2.25 ± 3.23 mL/15 min, and at third 2.25 ± 3.38 mL/15 min.  **Ocular:** Schirmer I test 9.86 ± 11.68 mm/5 min at first examination and 11.50 ± 14.92 mm/5 min at third; TBUT 10.79 ± 7.16 s at first examination, and 11.93 ± 9.71 s at third; Van Bijsterveld score 10.43 ± 4.73 at first examination and 9.04 ± 5.64 at third. |
| Pedersen, *et al.* [63], 1999 | Denmark | Case-control | pSjD patients, healthy age-matched controls, and reference group regarding possible age-related changes in the salivary flow rates and minor salivary gland morphology | - n=16, F 14/M 2   range 40-82 y   - n=14, F 13/M 1   range 39-70 y   - n=13, F 12/M 1 - range 20-33 y | **Oral:** self-reported (Beck’s inventory scale 0-4 and VAS, 0-100);  LSG biopsy, UWS (15 min),  **Ocular:** self-reported (Beck’s inventory scale 0-4 and VAS, 0-100) | **Oral:** 100% (n=16) a daily feeling of dry mouth, and 87.5% (n=14) frequently drank liquids to aid swallowing dry foods; pSjD median response 2/3 in the European classiﬁcation criteria of SjD; 68% (n=12) other symptoms, difﬁculty in chewing and speaking, need to drink water at night; 100% (n=16) experienced a dry mouth; 100% (n=16) with UWS<0.05 ml/min reported an annoying feeling of dry mouth that made speech difﬁcult; 0% of the young and age-matched healthy controls reported a feeling of dry mouth. 50% (n=8) of pSjD UWS <0.05 ml/min and 50% (n=8) UWS ≥0.05 ml/min.  **Ocular:** Symptoms of subjective dry eyes94% (n=15) pSjD; pSjD median response 2/3 in the European classiﬁcation criteria of SjD; 0% of the healthy controls experienced a daily feeling of dry eyes; 100% (n=16) pSjD diagnosed with KCS. |
| Rasker, *et al.* [71], 1990 | UK | Case-control | SCL patients with suspicion of SjD and three healthy control groups: otherwise healthy patients attending eyes clinics for routine prescription of spectacles, matched healthy controls for oral studies, matched healthy controls for sweat secretion | - n=26, F 22/M 4 53.5 ± 10.2 y - n=24, F/M NR  52.8 ± 10.3 y - n=24, F/M NR NR y - n=21, F/M NR 50 ± 12.2 y | **Oral:** self-reported;  stimulated submandibular flow (NR min), stimulated parotid flow (NR min), SGS (patients n=21),  **Ocular:** self-reported;  Rose Bengal staining Schirmer I test (5 min), Schirmer II test (NR min) (n=7) | **Oral:** 46.2% (n=12) patients and 25% (n=6) controls subjective dry mouth; 23.1% (n=6) patients and 12.5% (n=3) controls reported dry mouth as “mild”, 23.1% (n=6) patients and 0% (n=0) controls as “marked”, 7.7% (n=2) patients and 12.5% (n=3) controls as “severe”; 26.9% (n=7) patients an 16.7% (n=4) controls avoided dry food, 57.7% (n=15) patients an 25% (n=6) controls had difficulty swallowing, 19.2% (n=5) patients an 4.2% (n=1) controls had changed taste, 57.7% (n=15) patients an 50% (n=12) controls had dental prosthesis, 19.2% (n=5) patients and 4.2% (n=1) controls had problems with dental prosthesis, 23.1% (n=6) patients and 4.2% (n=1) controls had gland enlargement (episodic), 15.4% (n=4) patients and 0% controls had gland enlargement with pain and fever, 38.5% (n=10) patients and 29.2% (n=7) controls had hoarse voice, 19.2% (n=5) patients an 16.7% (n=4) controls had otherwise changed voice.  7.7% (n=2) patients had stimulated parotid and submandibular flow <50 µL/min. 14.3% (n=3) patients had altered SGS.  **Ocular:** Subjective dry eyes present in 34.6 % (n=9) patients and 33.3% (n=8) controls; 34.6% (n=9) patients and 4.2% (n=1) controls dry eyes, 19.2% (n=5) patients and 0% controls inability to cry on irritation or emotion, 19.2% (n=5) patients and 33.3% (n=8) controls burning sensation, 30.8% (n=8) patients and 45.8% (n=11) controls tiring soreness, 19.2% (n=5) patients and 0% controls pain, 38.5% (n=10) patients and 41.7% (n=10) controls itching, 23.1% (n=6) patients and 29.4% (n=7) controls had foreign body sensation, 23.1% (n=6) patients and 8.3% (n=2) controls redness, 34.6% (n=9) patients an 8.3% (n=2) controls excess of secretion, 11.5% (n=3) patients and 4.2% (n=1) controls difficulty in moving lids.  26.9% (n=7) patients and 4.2% (n=1) controls abnormal Schirmer I test; 85.7% (n=6) patients abnormal Schirmer II test; 7.7% (n=2/26) patients and 8.3% (n=2/24) controls abnormal Rose Bengal staining score; one patient symptom complex of dry eyes, abnormal Schirmer I test and abnormal Rose Bengal staining; 3.85% (n=1) patients and 4.2% (n=1) controls KCS. |
| Singh and Basu [70], 2022 | India | Case-control | SJS, SjD patients and healthy controls | - n=15, F/M NR   35.7 ± 10.9 y   - n=15, F/M NR   57.7 ± 19 y   - n=40, F/M NR   10-60 y   - split into groups: 20-30, 30-40, 40-50, 50-60 y | **Oral:** self-reported;  MSG count, MSG flow rate, LSG flow rate  **Ocular (SJS n=15, SjD n=15):** OSDI;  fluorescein staining, Schirmer I test (5 min) | **Oral:** 60% (n=9) SjD patients and 0% SJS patients subjective dry mouth; the salivary flow rate from lower labial glands in controls: 2.7 ± 0.29 μl/min in 20–30 y, 2.6 ± 0.39 μl/min in 30–40 y, 2.3 ± 0.6 μl/min in 40–50 y, and 1.9 ± 0.95 μl/min in 50–60 y; tmean n of secreting MSG openings in controls 38.7 ± 4.5 in 20-30 y, 37.2 ± 6.3 in 30-40 y, 30 ± 4.6 in 40-50 y and 22 ± 6.7 in 50-60 y; salivary flow rate from lower labial glands 0.5 ± 0.28 μl/min in SjD and 2.0 ± 0.95 μl/min in SJS; the mean n of secreting MSG openings 20.5 ± 5.9 in SJS and 12 ± 5.6 in SjD.  **Ocular**: OSDI 45.3 ± NR in SJS, 28.2 in SjD and (mean ± range) 6.2 ± 0-8 in controls.  The Schirmer I test (worse eye) 1.5 ± 2.1 mm/5 min in SjD, 4.8 ± 3.6 mm/5 min in SJS and (mean ± range) 22 ± 15-30 mm/5 min in controls; in SjD, OSS (mean ± range) 7.2 ± 4–11. |
| Smidt, *et al.* [64], 2011 | Denmark | Cross-sectional | Elderly population who had participated in the 4th examination of the Copenhagen City Heart Study | n=668, F 389/M 279  75.5 ± 6.4 y | **Oral:** self-reported;  unstimulated LSG flow rate (2 min), UWS (10 min), SWS (5 min),  **Ocular:** self-reported | **Oral:** 12.3% (n=82) subjective oral dryness. 23% (n=54/235) with oral dryness and 6.7% (n=28/421) with no oral dryness UWS ≤0.10 mL/min; 32.5% (n=13/40) with oral dryness and 11% (n=68/616) with no oral dryness SWS ≤0.70 mL/min.  **Ocular:** 10.7% (n=71) subjective ocular dryness. |
| Troncoso, *et al.* [65], 2024 | Spain | Cross-sectional | Sicca patients with SjD or non-SjD | n=142, F 127/M 15  56.1 ±1.5 y   - n=84, F 78/M 6   NR y   - n=58, F 49/M 9   NR y | **Oral:**  MSG biopsy, SGS, UWS (NR min)  **Ocular:**  Schirmer I test (NR min) | **Oral:** UWS 0.13 ± 0.19 mL/min, and 69% (n=98) UWS ≤0.1 mL/min; 78.6% (n=66) SjD and 55.2% (n=32) non-SjD UWS ≤0.1 mL/min; MSG biopsy abnormal in 90.9% (n=129); 95.1% (n=135) abnormal SGS results; the SGS qualitative evaluation 4.9% (n=7) as “mild”, 37.3% (n=53) “moderate”, 35.2% (n=50) “severe”, 14.8% (n=21) “functionally annulled”; the SGS qualitative evaluation in SjD 4.8% (n=4) “normal”, 4.8% (n=4) “mild”, 35.7% (n=30) “moderate”, 33.3% (n=28) “severe”, 21.8% (n=18) as “functionally annulled”; the SGS qualitative evaluation in non-SjD patients 5.2% (n=3) “normal”, 12.1% (n=7) “mild”, 39.7% (n=23) “moderate”, 37.9% (n=22) “severe”, 5.2% (n=3) “functionally annulled”. Abnormal time-activity curve detected in 19.1% (n=16) SjD and 6.9% (n=4) non-SjD; uptake ratio at parotid glands 110.24 cps/s for SjD and 119.08 cps/s for non-SjD; uptake ratio at submandibular glands 106.20 cps/s for SjD and 120.73 cps/s for non-SjD;gGlobal uptake ratio 108.22 cps/s for SjD and 119.91 cps/s for non-SjD; ejection fraction at parotid glands 37.9% in SjD and 42.9% in non-SjD; ejection fraction at submandibular glands 29.4% in pSjD and 33.1% in non-SjD; global ejection fraction 33.6% in SjD and 38% in non-SjD.  **Ocular:** Schirmer I test result 4.39 ± 5.68 mm, with 75.4% (n=107) Schirmer I test ≤5 mm/5 min; 81.0% (n=68) SjD and 67.2% (n=39) non-SjD Schirmer I test ≤5 mm/5 min. |
| Ture, *et al.* [66], 2023 | South Korea | Cross-sectional | pSjD patients | n=66, F 66/M 0  49.2 ± 11.3 y | **Oral:** self-reported (VAS, scale 0-10);  MSG biopsy (n=57), SGS of parotid and submandibular glands (n=64), UWS (15 min), SWS (10 min)  **Ocular:** self-reported (VAS, scale 0-10), OSDI (n=25);  OSS (n=51), Schirmer I test (5 min), TBUT (n=51) | **Oral: (median** ± **IQR)** Oral-VAS 7.0 ± 5.0-8.0;  UWS 0.01 ± 0.00–0.04 mL/min; SWS 0.22 ± 0.11–0.47 mL/min; parotid gland uptake ratio 2.5 ± 1.7–3.1 and parotid gland ejection fraction 40.7 ± 21.8–55.1%; submandibular gland uptake ratio 2.1 ± 1.8–2.5 and submandibular gland ejection fraction 14.5 ± 6.3–32.4%; MSG biopsy focus score 1.6 ± 0.8-25.  **Ocular: (median ± IQR)** Ocular-VAS 6.0 ± 4.0-7.0; OSDI 35.4 ± 25.0-58.3;  78.8% (n=52) Schirmer I test ≤5 mm/5 min; OSS 5.0 ± 2.0–10.0; 64.7% (n=33) OSS ≥4; TBUT 8.0 ± 6.0–11.0 s. |
| Villa and Abati [77], 2011 | Italy | Cross-sectional | Dental clinic attendees in Milan | n=601, F 204/M 296  (median ± IQR) 47 ± 32-63 y | **Oral:** self-reported (n=252)  **Ocular:** self-reported (n=252) | **Oral:** 31.7% (n=80) subjective dry mouth; 9.0% (n=54) a feeling of burning mouth; 13.1% (n=79) self-reported halitosis; 50.8% (n=128) dry lips; 37.7% (n=95) dry throat; 46.4% (n=117) waking up to drink water; 28.2% (n=71) were drinking water to facilitate swallowing.  **Ocular:** 26.6% (n=67) subjective dry eyes. |
| Wang, *et al.* [75], 2020 | Australia | Cross-sectional | Elderly population in South Australia | n=627, F 291/M 336  75 ± 7 y | **Oral:** S-XI  **Ocular:** self-reported | **Oral:** 21% (n=130) subjective dry mouth; S-XI 8.9 ± 3.1.  **Ocular:** 31% (n=205) subjective dry eye. In response to a question regarding frequency of dry eye symptoms, 53% (n=334) responded as “never”, 14% (n=88) “hardly ever”, 23% (n=145) “occasionally”, 7% (n=41) “fairly often” and 3% (n=19) “very often”. |
| Wangkaew, *et al.* [69], 2006 | Thailand | Case-control | Patients with RA, SLE, SCL and matched healthy controls for RA, SLE, SCL | - n=50, F 47/M 3   46.6 ± 8.8 y   - n=50, F 50/M 0   39.1 ± 9.6 y   - n=50, F 37/M 13   46.6 ± 7.9 y   - n=50; F 32/M 18  45.5 ± 8.3 y - n=50, F 36/M 14  39.1 ± 9.6 y - n=50, F 33/M 17 46.2 ± 7.3 y | **Oral:** self-reported;  SWS (2 min)  **Ocular:** self-reported;  Schirmer I test (5 min) | **Oral:** 6% (n=3) RA and 4% (n=2) controls ≥1 subjective oral symptoms; 22% (n=11) SLE and 0% controls ≥1 subjective oral symptoms; 16% (n=8) SCL and 4% (n=2) controls ≥1 subjective oral symptoms; 6% (n=3) RA and 0% controls xerostomia; 8% (n=4) SLE and 0% controls xerostomia; 14% (n=7) SCL and 0% controls xerostomia.  56% (n=28) of RA and 38% (n=19) controls pathological SWS; 50% (n=25) SLE and 32% (n=16) controls pathological SWS; 38% (n=19) SCL and 32% (n=16) controls pathological SWS; SWS in RA 2.8 ± 1.8 g, and controls 3.5 ± 1.7 g; SWS in SLE 2.8 ± 1.4 g, and controls 3.9 ± 1.8 g; SWS in SCL 3.5 ± 1.8 g, and controls 3.7 ± 1.7 g.  **Ocular:** 38% (n=14) RA patients and 18% (n=9) controls ≥1 subjective ocular symptoms; 36% (n=18) SLE and 14% (n=7) controls ≥1 subjective ocular symptoms; 54% (n=27) SCL and 16% (n=8) controls≥1 subjective ocular symptoms.  54% (n=27) RA and 22% (n=11) controls pathological Schirmer I test; 22% (n=11) SLE and 20% (n=10) controls pathological Schirmer I test; 32% (n=16) SCL and 32% (n=16) controls pathological Schirmer I test; Schirmer I test in right eye in RA 15.7 ± 16.4 mm, and controls 23.2 ± 16.4 mm; Schirmer I test in left eye in RA 13.6 ± 14.7 mm, and controls 20.7 ± 14.9 mm; Schirmer I test in right eye in SLE was 26.2 ± 17.9 mm, and controls 25.7 ± 15.5 mm; Schirmer I test in left eye in SLE 25.8 ± 17.4 mm, and controls 21.7 ± 15.3 mm. Schirmer I test in right eye in SCL 18.6 ± 16.6 mm, and controls 21.7 ± 16.3 mm; Schirmer I test in left eye in SCL 15.7 ± 14.7 mm, and controls 19.6 ± 16.1 mm; 18% (n=9) RA and 8% (n=4) of controls KCS; 18% (n=9) SLE and 2% (n=1) controls KCS; 18% (n=9) of SCL and 8% (n=4) controls KCS. |
| Wróbel-Dudzińska, *et al.* [67], 2021 | Poland | Case-control | Population from Lublin district area attending dental and optometrist clinics, split into six groups based on their general health: A (controls), B-F (patients) | n=642, F 405/M 237  50.2 ± 11.4 y   - A: n=156, F 92/M 64  45.1 ± 12.2 y - B: n=109, F 67/M 42  49 ± 11.1 y - C: n=103, F 86/M 17  50 ± 10.7 y - D: n=115, F 78/M 37  52.3 ± 8.4 y - E: n=84, F 45/M 39   51.7 ± 9.2 y   - F: n=75, F 37/M 38   57.7 ± 12.1 y | **Oral:** self-reported;  parotid flow (Schirmer test - in front of parotid gland duct, 1 min), UWS (Schirmer test - floor of mouth, 1 min)  **Ocular:** self-reported;  fluorescein staining, Schirmer I test (5 min) | **Oral:** 35.8% (n=230) subjective mouth dryness; 23.36% (n=150) burning sensation; 31% (n=199) need for moisturizing the lips; 6.07% (n=39) eat sweets and chewing gum to stimulate saliva production.  UWS 18.56 ± 1.79 mm/min in A, 14.31 ± 1.48 mm/min in B, 12.34 ± 1.83 mm/min in C, 15.34 ± 1.56 mm/min in D, 12.47 ± 1.83 mm/min in E, 11.00 ± 1.79 mm/min in F; parotid flow 26.85 ± 2.81 mm/min in A, 20.16 ± 2.33 mm/min in B, 16.61 ± 2.39 mm/min in C, 21.57 ± 1.96 mm/min in D, 17.41 ± 2.71 mm/min in E, 14.5 ± 2.38 mm/min in F.  **Ocular:** 26.0% (n=167) foreign body sensation. 16.8% (n=108) itching; 18.3% (n=118) burning eyes; Schirmer I test 18.61 ± 2.23 mm/5 min in A, 14.53 ± 1.87 mm/5 min in B, 12.47 ± 1.65 mm/5 min in C, 14.68 ± 1.66 mm/5 min in D, 12.39 ± 1.70 mm/5 min in E and 9.55 ± 1.29 mm/5 min in F; no participants Schirmer I test results ≤5 mm; Schirmer I test 6-10 mm in: C (n=12), D (n=2), E (n=12) and F (n=56). Results of 11-15 mm were in: A (n=17), B (n=72), C (n=91), D (n=74), E (n=69) and F (n=19). Results of ≥16 mm were in: A (n=138), B (n=37), D (n=39), and E (n=3). |
| Xin, *et al.* [68], 2020 | Hong Kong | Case-control | pSjD, aSjD patients and healthy controls | - n=38, F 36/M 2   50.8 ± 8.5 y   - n=47, F 47/M 0   50 ± 10.5 y   - n=40, F 40/M 0 - 51.4 ± 7.4 y | **Oral:** SSI oral domain score;  UWS (5 min), SWS (5 min)  **Ocular:** SSI ocular domain score | **Oral:** 92.1% (n=35) pSjD, 80.9% (n=38), aSjD and 12.5% (n=5) controls difficulty eating. 89.5% (n=34) pSjD, 87.2% (n=41) aSjD and 32.5% (n=13) controls dry throat or nose; 73.7% (n=28) pSjD, 74.5% (n=25) aSjD and 50.0% (n=20) controls bad breath; 86.8% (n=33) pSjD, 87.2% (n=41) aSjD and 25% (n=10) controls wetting mouth; 63.2% (n=24) pSjD, 57.4% (n=27) aSjD and 25% (n=10) controls other oral problems; SSI oral domain score 2.7 ± 1.5 for pSjD, 2.6 ± 1.6 for aSjD and 0.7 ± 0.9 for controls.  UWS 0.2 ± 0.2 mL/min for pSjD, 0.3 ± 0.2 mL/min for aSjD and 0.5 ± 0.3 mL/min for controls; SWS 0.4 ± 0.5 mL/min for pSjD, 0.5 ± 0.5 mL/min for aSjD and 1.1 ± 0.6 mL/min for controls.  **Ocular:** 84.5% (n=32) pSjD, 85.1% (n=40) aSjD and 37.5% (n=15) controls sore eyes; 94.7% (n=36) pSjD, 91.5% (n=43) aSjD and 67.5% (n=27) controls eye irritation; 63.2% (n=24) pSjD, 80.9% (n=42) aSjD and 45.0% (n=18) controls poor vision; SSI ocular domain score 2.8 ± 1.6 for pSjD, 2.9 ± 1.7 for aSjD and 1.5 ± 1.4 for controls. |

Abbreviations: ^131^I-activity – activity after vectorized internal radioactive iodine therapy, cGVHD – chronic graft-versus-host disease, CODS – clinical oral dryness, DED-VAS – dry eye disease symptoms visual analogue scale, DTC – differentiated thyroid carcinoma, F – female, HPCT – haematopoietic progenitor cell transplantation, KCS – keratoconjunctivitis sicca, LSG – labial salivary gland (reported as LMSG), M – male, MDEQ – McMonnies dry eye questionnaire, MG – meibomian gland, MSG – minor salivary gland, NR – not reported, OSDI – ocular surface disease index (0-100), OSS – Ocular Staining Score (0-12), pSjD – primary Sjögren’s disease (reported as pSS), RA – rheumatoid arthritis, SCL – systemic sclerosis SGS – salivary gland scintigraphy, SJS – Stevens-Johnson syndrome, SLE – systemic lupus erythematosus, SjD – Sjögren’s disease (also known as SS), aSjD – associated Sjögren’s disease (reported as sSS), SWS – stimulated whole saliva (reported as SWFS, SWSFR, SFR, Saxon(’s) test), S-XI – summated xerostomia inventory (reported as Summated Xerostomia Inventory-Dutch Version), TBUT – tear break up time (reported as FBUT, TFBUT, BUT), UWS – unstimulated whole saliva (reported as UWSF, UWSFR, UFR), XI – xerostomia inventory, y – years.

**Table S3.** Study quality assessment of observational cohort and cross-sectional studies based on NIH “Quality Assessment Tool for Observational Cohort and Cross-Sectional Studies”.

| **Study**  **Criteria** | **Bassim, et al. [53]** | **Baudin, *et al.* [54]** | **Bergdahl [55]** | **Bezzina, *et al.* [56]** | **Billings, *et al.* [57]** | **Błochowiak [58]** | **Da Cunha, *et al.* [59]** | **Fernandez Castro, *et al.* [73]** | **Hynne, *et al.* [61]** | **Kalk, *et al.* [52]** | **Kasetsuwan, *et al.* [76]** | **Lackner, *et al.* [62]** | **Oxholm, *et al.* [51]** | **Smidt, *et al.* [64]** | **Troncoso, *et al.* [65]** | **Ture, *et al.* [66]** | **Villa and Abati [77]** | **Wang, *et al.* [75]** |
| --- | --- | --- | --- | --- | --- | --- | --- | --- | --- | --- | --- | --- | --- | --- | --- | --- | --- | --- |
| 1. Was the research question or objective in this paper clearly stated? | YES | YES | YES | YES | YES | YES | YES | YES | YES | YES | YES | YES | YES | YES | YES | YES | YES | YES |
| 2. Was the study population clearly specified and defined? | NO | YES | YES | YES | NO | NO | YES | YES | YES | YES | NO | NO | NO | YES | YES | NO | YES | YES |
| 3. Was the participation rate of eligible persons at least 50%? | NR | NR | YES | YES | NR | NR | NR | NR | NO | NR | NO | NR | NR | NO | NR | NR | NR | NR |
| 4. Were all the subjects selected or recruited from the same or similar populations (including the same time period)? Were inclusion and exclusion criteria for being in the study prespecified and applied uniformly to all participants? | YES | YES | YES | YES | NO | NR | YES | YES | YES | YES | YES | YES | NO | YES | YES | NR | YES | YES |
| 5. Was a sample size justification, power description, or variance and effect estimates provided? | NO | YES | NO | NO | NO | NO | NO | NO | NO | NO | NO | NO | NO | NO | NO | NO | NO | NO |
| 6. For the analyses in this paper, were the exposure(s) of interest measured prior to the outcome(s) being measured? | YES | YES | NO | NO | NO | YES | NO | NO | NO | NO | NO | NO | YES | NO | NO | NO | NO | NO |
| 7. Was the timeframe sufficient so that one could reasonably expect to see an association between exposure and outcome if it existed? | YES | YES | NR | NR | NR | NR | NR | NR | NR | NR | NR | NR | YES | NR | NR | NR | NR | NR |
| 8. For exposures that can vary in amount or level, did the study examine different levels of the exposure as related to the outcome (e.g., categories of exposure, or exposure measured as continuous variable)? | YES | YES | YES | NA | YES | NO | NA | YES | NA | YES | YES | NA | NA | NO | YES | NA | NA | NA |
| 9. Were the exposure measures (independent variables) clearly defined, valid, reliable, and implemented consistently across all study participants? | YES | YES | YES | YES | YES | YES | YES | YES | YES | YES | NO | YES | NO | NO | YES | YES | NO | YES |
| 10. Was the exposure(s) assessed more than once over time? | NO | YES | NO | NO | NO | NO | NO | NO | NO | NO | NO | NO | YES | NO | NO | NO | NO | NO |
| 11. Were the outcome measures (dependent variables) clearly defined, valid, reliable, and implemented consistently across all study participants? | NO | YES | NO | YES | NO | YES | YES | YES | YES | YES | NO | YES | YES | NO | YES | YES | NO | YES |
| 12. Were the outcome assessors blinded to the exposure status of participants? | NO | NO | NO | NO | NO | NO | NO | NO | NO | NO | NO | YES | NO | NO | NO | NO | NA | NA |
| 13. Was loss to follow-up after baseline 20% or less? | NA | YES | NA | NA | NA | NA | NA | NA | NA | NA | NA | NA | NR | NA | NA | NA | NA | NA |
| 14. Were key potential confounding variables measured and adjusted statistically for their impact on the relationship between exposure(s) and outcome(s)? | YES | YES | YES | YES | YES | NO | NO | YES | YES | NO | YES | NO | NO | YES | YES | NO | YES | NO |
| **Final rating** | Fair | Good | Fair | Fair | Poor | Poor | Fair | Fair | Fair | Fair | Poor | Fair | Fair | Poor | Fair | Poor | Fair | Fair |

Abbreviations: NA – not applicable, NR – not reported

**Table S4.** Study quality assessment of case-control studies based on NIH “Quality Assessment of Case-Control Studies”.

| **Study**  **Criteria** | **Chiu, *et al.* [72]** | **Gilboe, *et al.* [60]** | **Leite, *et al.* [74]** | **Pedersen, *et al.* [63]** | **Rasker, *et al.* [71]** | **Singh and Basu [70]** | **Wangkaew, *et al.* [69]** | **Wrobel-Dudzinska, *et al.* [67]** | **Xin, *et al.* [68]** |
| --- | --- | --- | --- | --- | --- | --- | --- | --- | --- |
| 1. Was the research question or objective in this paper clearly stated and appropriate? | YES | YES | YES | YES | YES | YES | YES | YES | YES |
| 2. Was the study population clearly specified and defined? | NO | YES | NO | NO | NO | NO | NO | NO | NO |
| 3. Did the authors include a sample size justification? | NO | NO | NO | NO | NO | NO | NO | NO | YES |
| 4. Were controls selected or recruited from the same or similar population that gave rise to the cases (including the same timeframe)? | YES | NO | NO | NO | NO | NO | NO | NO | YES |
| 5. Were the definitions, inclusion and exclusion criteria, algorithms or processes used to identify or select cases and controls valid, reliable, and implemented consistently across all study participants? | NO | YES | NO | NO | NO | YES | YES | NO | YES |
| 6. Were the cases clearly defined and differentiated from controls? | YES | YES | YES | YES | YES | YES | YES | NO | YES |
| 7. If less than 100 percent of eligible cases and/or controls were selected for the study, were the cases and/or controls randomly selected from those eligible? | NR | NR | NO | NR | NR | NR | NR | NR | NR |
| 8. Was there use of concurrent controls? | NO | NO | NO | NO | NO | NO | NO | NO | NO |
| 9. Were the investigators able to confirm that the exposure/risk occurred prior to the development of the condition or event that defined a participant as a case? | NR | YES | NR | NR | NR | NR | NR | NR | NR |
| 10. Were the measures of exposure/risk clearly defined, valid, reliable, and implemented consistently (including the same time period) across all study participants? | YES | YES | NO | NO | NO | NO | YES | YES | YES |
| 11. Were the assessors of exposure/risk blinded to the case or control status of participants? | NO | NO | NO | NO | NO | NO | NO | NO | NO |
| 12. Were key potential confounding variables measured and adjusted statistically in the analyses? If matching was used, did the investigators account for matching during study analysis? | YES | YES | YES | NO | NR | NO | NO | NO | NO |
| **Final rating** | Fair | Fair | Poor | Poor | Poor | Poor | Poor | Poor | Fair |

Abbreviations: NA – not applicable, NR – not reported

**Table S5.** Matrix of objective and subjective oral and ocular assessments in the included studies, among which correlation and/or association analysis was performed. Bolded reference indicates at least one significant correlation between the assessments (sometimes multiple correlations were carried out, for example due to different patient groups). For non-significant results, only the available numerical values were included in this table.

|  | | *N studies with significant results/total N* | *Ref.* | | | *N studies with significant results/total N* | *Ref.* | | | *N studies with significant results/total N* | *Ref.* | | *N studies with significant results/total N* | | *Ref.* | | *N studies with significant results/total N* | | *Ref.* | *N studies with significant results/total N* | *Ref.* | | | *N studies with significant results/total N* | *Ref.* | | | *N studies with significant results/total N* | *Ref.* | | *N studies with significant results/total N* | | *Ref.* | *N studies with significant results/total N* | | *Ref.* | *N studies with significant results/total N* | *Ref.* | |
| --- | --- | --- | --- | --- | --- | --- | --- | --- | --- | --- | --- | --- | --- | --- | --- | --- | --- | --- | --- | --- | --- | --- | --- | --- | --- | --- | --- | --- | --- | --- | --- | --- | --- | --- | --- | --- | --- | --- | --- |
| **Ocular assessments**  **Oral assessments** | | **OBJECTIVE** | | | | | | | | | | | | | | | | | | **SUBJECTIVE** | | | | | | | | | | | | | | | | | **OBJECTIVE AND SUBJECTIVE** | | |
|  |  | **Schirmer I test** | | | **TBUT** | | | | **OSS** | | | **van Bijsterveld score** | | | | **presence/diagnosis of KCS symptoms** | | | | **OSDI** | | | **MDEQ** | | | | **SSI ocular domain score** | | | | | **self-reported symptoms/questionnaires** | | | | | **ocular sensitivity** | | |
| **OBJECTIVE** | **UWS** | 2/5 | | [**51**, 59–61, **67**] | 0/3 | | | [59, 61, 66] | 0/3 | | [59, 61, 66] | 1/1 | | **[51]** | | 0/1 | | [59] | | 1/3 | | [54, 61, **62**] | 0/1 | | | [61] | 1/1 | | | **[68]** | | 2/6 | | | [54, **58**, 62**–**64, 66] | | NR | | |
|  |  |  |  |  |  |  |  |  |  |  |  |  |  |  |  |  |  |  |  |  |  |  |  |  |  |  |  |  |  |  |  |  |  |  |  |  |  |  |  |
|  | **SWS** | 1/2 | | [61, **69**] | 0/2 | | | [61, 66] | 1/2 | | [61, **66**] | NR | | | | 1/1 | | **[69]** | | 1/3 | | [54, 61, **62**] | 0/1 | | | [61] | 1/1 | | | **[68]** | | 0/5 | | | [54, 62, 64, 66, 69] | | NR | | |
|  |  |  |  |  |  |  |  |  |  |  |  |  |  |  |  |  |  |  |  |  |  |  |  |  |  |  |  |  |  |  |  |  |  |  |  |  |  |  |  |
|  | **parotid gland flow rate** | 1/1 | | **[67]** | NR | | | | NR | | | NR | | | | NR | | | | NR | | | NR | | | | NR | | | | | NR | | | | | NR | | |
|  |  |  |  |  |  |  |  |  |  |  |  |  |  |  |  |  |  |  |  |  |  |  |  |  |  |  |  |  |  |  |  |  |  |  |  |  |  |  |  |
|  | **submandibular/sublingual glands flow rate** | 1/1 | | **[52]** | NR | | | | NR | | | NR | | | | NR | | | | NR | | | NR | | | | NR | | | | | NR | | | | | NR | | |
|  |  |  |  |  |  |  |  |  |  |  |  |  |  |  |  |  |  |  |  |  |  |  |  |  |  |  |  |  |  |  |  |  |  |  |  |  |  |  |  |
|  | **MSG flow rate** | 0/1 | | [70] | NR | | | | NR | | | NR | | | | NR | | | | NR | | | NR | | | | NR | | | | | NR | | | | | NR | | |
|  |  |  |  |  |  |  |  |  |  |  |  |  |  |  |  |  |  |  |  |  |  |  |  |  |  |  |  |  |  |  |  |  |  |  |  |  |  |  |  |
|  | **LSG flow rate** | NR | | | NR | | | | NR | | | NR | | | | NR | | | | NR | | | NR | | | | NR | | | | | 0/1 | | | [64] | | NR | | |
|  |  |  |  |  |  |  |  |  |  |  |  |  |  |  |  |  |  |  |  |  |  |  |  |  |  |  |  |  |  |  |  |  |  |  |  |  |  |  |  |
|  | **LSG biopsy** | NR | | | NR | | | | NR | | | NR | | | | NR | | | | NR | | | NR | | | | NR | | | | | 1/2 | | | [73, **74**] | | NR | | |
|  | **MSG biopsy** | NR | | | 0/1 | | | [66] | 1/1 | | **[66]** | NR | | | | NR | | | | NR | | | NR | | | | NR | | | | | 0/1 | | | [66] | | NR | | |
|  | **SGS** | 0/2 | | [65, 72] | 0/1 | | | [66] | 1/1 | | **[66]** | NR | | | | NR | | | | NR | | | NR | | | | NR | | | | | 0/1 | | | [66] | | NR | | |
| **SUBJECTIVE** | **XI** | 0/1 | | [62] | NR | | | | NR | | | NR | | | | NR | | | | NR | | | NR | | | | NR | | | | | NR | | | | | NR | | |
|  | **S-XI** | 0/1 | | [61] | 0/1 | | | [61] | 0/1 | | [61] | NR | | | | NR | | | | 1/1 | | **[61]** | 1/1 | | | **[61]** | NR | | | | | 1/1 | | | **[75]** | | NR | | |
|  | **presence/diagnosis of xerostomia symptoms** | 2/2 | | **[61, 69]** | 0/1 | | | [61] | 1/1 | | **[61]** | NR | | | | 1/1 | | **[69]** | | 1/1 | | **[61]** | 1/1 | | | **[61]** | NR | | | | | 3/3 | | | **[57, 69, 75]** | | NR | | |
|  | **self-reported symptoms/questionnaires** | 2/3 | | [62, **69, 71]** | 0/1 | | | [66] | 0/1 | | [66] | NR | | | | 1/1 | | **[69]** | | 0/1 | | [66] | NR | | | | NR | | | | | 10/10 | | | **[53–55, 64, 66, 69, 71, 74, 76, 77]** | | NR | | |
| **OBJECTIVE AND SUBJECTIVE** | **oral sensitivity** | NR | | | NR | | | | NR | | | NR | | | | NR | | | | NR | | | NR | | | | NR | | | | | NR | | | | | 1/1 | | **[56]** |

Abbreviations: KCS - keratoconjunctivitis sicca, LSG - labial salivary gland, MDEQ - McMonnies Dry Eye Questionnaire, MSG - minor salivary gland, NR – no statistics for associations and/or correlations reported, OSDI - Ocular Surface Disease Index, OSS - ocular staining score, SGS - salivary gland scintigraphy, SWS - stimulated whole saliva, S-XI - Summated Xerostomia Inventory, TBUT - tear break-up time, UWS - unstimulated whole saliva, XI - Xerostomia Inventory
